# Tunable optical and UV-blocking properties of eco-friendly CMC/ZnO nanocomposite films

**DOI:** 10.1038/s41598-026-58695-5

**Published:** 2026-07-12

**Authors:** Rania Badry, Mahmoud M. El-Nahass, Nadra Nada, Hanan Elhaes, Medhat A. Ibrahim

**Affiliations:** 1 Physics Department, Faculty of Women for Arts, Science and Education, Ain Shams University, Cairo, 11757 Egypt; 2 Physics Department, Faculty of Education, Ain Shams University, Roxy, Cairo, Egypt; 3 Spectroscopy Department, National Research Centre, 33 El-Bohouth St, Dokki, Giza 12622 Egypt; 4 Molecular Modeling and Spectroscopy Laboratory, Centre of Excellence for Advanced Science, National Research Centre, 33 El-Bohouth St., Dokki, Giza 12622 Egypt; 5 Center for Converging Sciences and Emerging Technologies (CoSET), Benha National University (BNU), El-Obour, 13518 Egypt

**Keywords:** ZnO, CMC, Optical band gap, Refractive index, UV blocking capacity, Green chillies, Chemistry, Environmental sciences, Materials science, Nanoscience and technology

## Abstract

As environmental sustainability standards become more stringent, there is a growing need for biodegradable and renewable UV-protective films. Thus, this study looked at how zinc oxide nanoparticles (ZnO-NPs) affect the optical properties of carboxymethyl cellulose sodium (CMC) using a standard method called solution-casting. Fourier-transform infrared (FTIR) spectroscopy revealed that the carboxyl groups of CMC were ionized, forming hydrogen bonds with ZnO-NPs. X-ray diffraction (XRD) analysis confirmed the formation of CMC/ZnO nanocomposites through the characteristic diffraction peaks of ZnO-NPs, while the crystallite size of the nanocomposites decreased with increasing ZnO-NP concentration. The optical analysis found that as the amount of ZnO increased, the HOMO/LUMO band gap decreased from 5.21 eV to 5.10, 4.67, 4.43, and 3.98 eV, which was linked to the smaller crystallite size. Moreover, the refractive index of CMC increased from 1.78 to 1.84, 2.23, 2.51, and 2.60 due to the addition of 2, 4, 6, and 8  wt.% of ZnO-NPs, indicating modified optical properties suitable for various applications. The CMC/ZnO nanocomposite films showed strong UV-blocking performance, with the film containing 8 wt% ZnO-NPs blocking 94% of UVC (200–280 nm), 93.5% of UVB (280–320), and 93% of UVA (320–400 nm) radiation. Thus, based on the known biodegradable nature of the CMC matrix, CMC/ZnO nanocomposites can be considered as promising candidates for biodegradable UV-protective materials. The long-term stability of the optical properties was evaluated by re-measuring the absorbance and transmittance of pure CMC films and those doped with 8 wt% ZnO-NPs after more than 6 months of storage under normal conditions. Despite minor variations in film thickness, the optical measurements remained highly consistent, demonstrating that both pure and doped CMC films retain their optical performance over extended periods. A preliminary test using green chillies was conducted to evaluate the moisture-retention performance of the prepared films under UVA light, providing an initial indication of their ability to reduce water loss and maintain firmness during storage.

## Introduction

In recent decades, increasing worldwide awareness of environmental degradation and excessive dependence on petroleum-based polymers have spurred significant initiatives aimed at developing sustainable, biodegradable, and renewable materials^1–3^. The proliferation of non-biodegradable plastics in the environment presents significant ecological and health risks. As a result, researchers have been compelled to investigate natural polymer-based alternatives that integrate environmental sustainability with advantageous functional characteristics^4–6^. Cellulose and its derivatives are potential biopolymers due to their abundance, renewability, and superior film-forming properties^7,8^.

Sodium carboxymethyl cellulose (CMC), a chemically altered version of cellulose, has gained significant interest due to its solubility in water, non-toxic nature, transparency, and outstanding film-forming properties^9^. Due to these features, CMC has been extensively used in areas like food packaging, pharmaceutical coatings, and optical films. However, pure CMC films show specific limitations, including low mechanical strength, excessive hydrophilicity, and inadequate resistance to ultraviolet (UV) light, which greatly limit their real-world uses. To address these constraints, integrating inorganic nanoparticles into CMC matrices has been acknowledged as an effective strategy to improve their structural, mechanical, and optical properties^10–13^.

The remarkable physicochemical features of zinc oxide nanoparticles (ZnO-NPs)—including their high UV absorption capacity, robust photocatalytic activity, superior thermal and chemical stability, and antibacterial behavior have led to extensive investigation into their potential use as functional fillers in polymeric systems^14^. In addition,  ZnO is a desirable additive to produce biodegradable nanocomposites due to its low cost, lack of environmental impact, and general biocompatibility^15–17^. ZnO is a great reinforcing ingredient for nanocomposites made of CMC because of these qualities. Strong interfacial interactions, including electrostatic attraction or hydrogen bonding, between the carboxyl and hydroxyl groups of CMC and the surface of ZnO-NPs can occur when ZnO-NPs are incorporated into CMC matrices. The interactions mentioned above have the potential to greatly impact the films’ structural organization, optical behavior, and energy band structure^18–20^.

Adjusting the concentration of nanoparticles enables precise alterations to the optical band gap and UV-shielding capabilities, facilitating the creation of multifunctional materials tailored for targeted applications^21^. The creation of these nanocomposite films therefore signifies a promising approach for developing biodegradable coatings and packaging materials that offer UV protection while enhancing optical and environmental stability^22^.

Our research group has continuously explored both experimental and theoretical approaches to enhance the optical and electronic performance of CMC. In recent years, numerous studies have investigated the impact of adding various inorganic oxides and carbon-based nanomaterials to the CMC matrix to improve their performance for optoelectronic and sensing purposes^22^. For instance, Badry et al.^23^ performed molecular modeling analyses to investigate the structural behavior of CMC treated with acetic acid, providing fundamental insights into its molecular interactions and modification pathways. Later, Badry et al.^24^ conducted a theoretical study on CMC/ZnO nanocomposites and demonstrated their potential as gas sensors for NH₃, H₂S, and HBr detection. Building on these findings, Badry et al.^25^ investigated the UV-blocking and structural characteristics of nanocomposites based on CMC and CuO highlighting their strong UV-shielding efficiency. In a related work, Badry et al.^26^ incorporated core/shell nanoparticles (CuO@ZnO) into the CMC matrix, achieving films with superior UV-filtering capability and a high refractive index suitable for optical device applications.

More recently, Badry et al.^27^ reported the enhancement of the optoelectronic characteristics of CMC through the incorporation of ZnO/graphene oxide (GO) and CuO/GO nanocomposites, emphasizing their potential in antimicrobial and packaging applications. Aldaleeli et al.^28^ examined the effect of varying graphene concentrations on the structural and optical properties of CMC, illustrating the adjustability of the band gap with carbon-based nanofillers^24^. Finally, these findings illustrate our group’s systematic efforts to tailor the optical characteristics of CMC-based materials by a combination of theoretical modeling and experimental synthesis.

This study involved the preparation of CMC/ZnO nanocomposite films via the usual solution-casting method, followed by a comprehensive investigation of their structural and optical properties. Fourier-transform infrared (FTIR) spectroscopy and X-ray diffraction (XRD) were utilized to clarify the molecular interactions between CMC and ZnO-NPs and to verify the synthesis of the nanocomposites. The optical properties, such as band gap energy and refractive index, were examined in relation to the concentration of ZnO-NPs. The primary objective is to demonstrate the strong UV-blocking capability, particularly at higher ZnO loadings of CMC/ZnO nanocomposites, where the films effectively attenuated UVC, UVB, and UVA radiation. Based on the intrinsic biodegradable nature of the CMC polymer, the CMC/ZnO nanocomposites may serve as promising biodegradable UV-protective materials; however, biodegradability was not experimentally evaluated within the scope of this work.

## Experimental section

### Chemicals

The compounds and their purity (sodium hydroxide pellets, pure ethanol, dehydrated zinc acetate, CMC) were consistent with those documented in our prior work^26^. All reagents were utilized without additional purification. Sodium hydroxide pellets and pure ethanol (analytical grade, 99.9%) were procured from El Nasr Pharmaceutical Chemicals Co. (Cairo, Egypt). Dehydrated zinc acetate (laboratory reagent, 99%) was acquired from Sigma-Aldrich (Germany). Carboxymethyl cellulose sodium (CMC) having a molecular weight of 2.5 × 10^5^ g/mole was procured from K. Patel Chemo Pharma PVT (India). All glassware was meticulously cleansed with a soap solution and subsequently rinsed with deionized water before utilization.

### ZnO preparation

The ZnO-NPs were synthesized utilizing the identical methodology employed in our previous research for the preparation of the ZnO component of the CuO@ZnO core/shell nanoparticles^26^, with minor modifications in the concentration of the precursor, hence assuring uniformity in synthesis conditions and particle attributes.

 ZnO nanoparticles were synthesized via the precipitation process. Initially, 0.2 mol of zinc acetate was solubilized in 100 mL of deionized water and agitated quickly with a magnetic stirrer until fully dissolved. The solution was subsequently heated to 60 °C for 30 min. Independently, 0.4 mol of NaOH was solubilized in 50 mL of deionized water. The NaOH solution was incrementally added to the zinc acetate solution while being vigorously stirred for 3 h, leading to the emergence of a white precipitate.

The precipitate was repeatedly rinsed with deionized water, followed by centrifugation after each washing step to ensure thorough removal of soluble precursors and by-products. In the final purification step, the sample was washed with ethanol and centrifuged again to enhance the removal of any remaining organic residues. The obtained solid was then filtered and dehydrated in an oven at 80 °C for 2.5 h. Finally, the dried material was calcined at 500 °C for 2 h to eliminate residual hydroxyl and carbonyl groups, yielding purified ZnO-NPs^25^. The ZnO-NPs were synthesized utilizing the identical methodology employed in our previous research for the preparation of the ZnO component of the CuO@ZnO core/shell nanoparticles, hence assuring uniformity in synthesis conditions and particle attributes^22^.

### CMC/ZnO nanocomposite film preparation

The concentrations of ZnO-NPs were chosen to be identical to those employed for CuO nanoparticles in our previous work^25^, in order to enable a systematic comparison of the changes in the optical properties of CMC induced by the incorporation of either CuO or ZnO. Additionally, CMC films and CMC doped with different concentrations of ZnO-NPs were prepared using the solution-casting method as previously reported for the preparation of CMC/CuO nanocomposites^25^. Initially, 0.5 g of CMC was dissolved in 50 mL of deionized water and stirred with a magnetic stirrer for 2 h at 50 °C until completely dissolved. Different concentrations of ZnO-NPs (2, 4, 6, and 8 wt%) were then added to the CMC solution and stirred continuously for an additional 2 h. The samples were labeled according to the ZnO-NPs content, as presented in Table 1.

In this work, the ZnO content was limited to 8 wt% because higher concentrations showed, in preliminary experiments, a clear reduction in dispersion within the CMC matrix, which resulted in particle agglomeration and difficulty in achieving uniform films. These defects influenced the optical quality of the samples, thus concentrations higher than 8 wt% were not considered in the present study.

All solutions containing CMC/ZnO nanocomposite were treated with ultrasonic waves for 1 h in order to prevent the agglomeration of ZnO-NPs. The solutions were subsequently poured into plastic Petri dishes and dried at 40 °C for approximately 24 h. The resulting films were carefully peeled from the dishes. Using a digital micrometer gauge, the thickness of the films made of pure CMC and nanocomposite were measured. Table 1 summarizes the thicknesses of the nanocomposite films, while the pure CMC film had a thickness of 0.032 mm.

In the solution-casting method, film thickness is primarily influenced by factors such as solution volume, casting area, polymer concentration, nanoparticles loading, and drying conditions. Although we maintained a fixed casting volume and drying environment to achieve comparable films, the incorporation of nanoparticles affects solution viscosity and distribution, making it difficult to obtain perfectly identical thickness values across all samples. Therefore, slight variations in thickness among the nanocomposite films are expected and commonly reported for cast films. These variations in the film-thickness may contribute to minor differences in optical transmittance.

**Table 1:** Sample coding, ZnO-NPs concentration, and thickness of CMC and CMC/ZnO nanocomposite films.

Sample	CMC (g)	ZnO (wt.%)	D.W. (mL)	Thickness (mm)
CMC	0.5	0		0.032
A1		2		0.036
A2		4	50	0.028
A3		6		0.026
A4		8		0.023

### Characterization techniques

The X-ray diffraction (XRD) pattern of ZnO powder was measured separately over a 2θ range of 25°–80°, while the CMC/ZnO nanocomposite samples were obtained in the 2θ range of 5–90° utilizing a Philips X-ray diffractometer with a step size of 0.026°. A Cu Kα radiation source (λ = 1.54 Å) operated at 45 kV and 40 mA was employed to examine the crystalline structure. Powdered samples were made by manually grinding with a mortar and pestle in a minimal quantity of ethanol to reduce sample loss and avert structural damage. XRD measurements were performed at the Nanotechnology Research Centre (NTRC), British University, Egypt, under regulated laboratory conditions to guarantee measurement precision and repeatability.

The Fourier-transform infrared spectroscopy (FTIR, Bruker Vertex 70) in the 4000–400 cm^−1^ wavenumber region was used to study the molecular structure of CMC and ZnO-NPs. A type IIa diamond crystal and a diamond ATR accessory with a 2 μm penetration depth were utilized. The spectra were captured using 35 scans per sample at a resolution of 4 cm^−1^. Air was used to record background spectra under the same settings. No additional steps were taken to prepare any of the samples for analysis.

A double-beam UV-Vis-NIR spectrophotometer, namely a Jasco model, was used to record the optical absorption spectra within the 200–1000 nm wavelength range. In order to guarantee the precision and repeatability of the measurements, FTIR and UV-Vis-NIR were carried out in a controlled laboratory setting at the Institute of Physics Research, National Research Centre (NRC), Egypt. Note that all optical measurements in this study were performed using solid CMC/ZnO nanocomposite films, not solution-based samples.

Additionally, optical measurements were collected from multiple positions on each film to ensure that the recorded data were representative of the overall sample area. These repeated measurements showed only minor variations, confirming that the films possessed sufficient spatial uniformity for reliable optical analysis. In addition, controlled casting conditions were maintained throughout the preparation process to minimize thickness irregularities and promote homogeneous film formation.

The packaging experiment was carried out in accordance with the procedure described by Li et al.^29^. Fresh green chilli was obtained from local fruit and vegetable markets in Giza, Egypt. The chillies were washed thoroughly with distilled water and subsequently dried. Only disease-free green chillies of similar maturity, but varying in size, were selected for the packaging process.

The chillies were assigned at random to three experimental groups. The first group functioned as the non-packaged control. The second group was enclosed in CMC/6wt.% ZnO film, while the third group was packaged using CMC/8wt.% ZnO film. Each packaging treatment was prepared in three replicates. All groups were stored at an ambient temperature of 23 °C for 3 days under UVA lamp.

## Results and discussion

### FTIR results

**Fig. 1:**
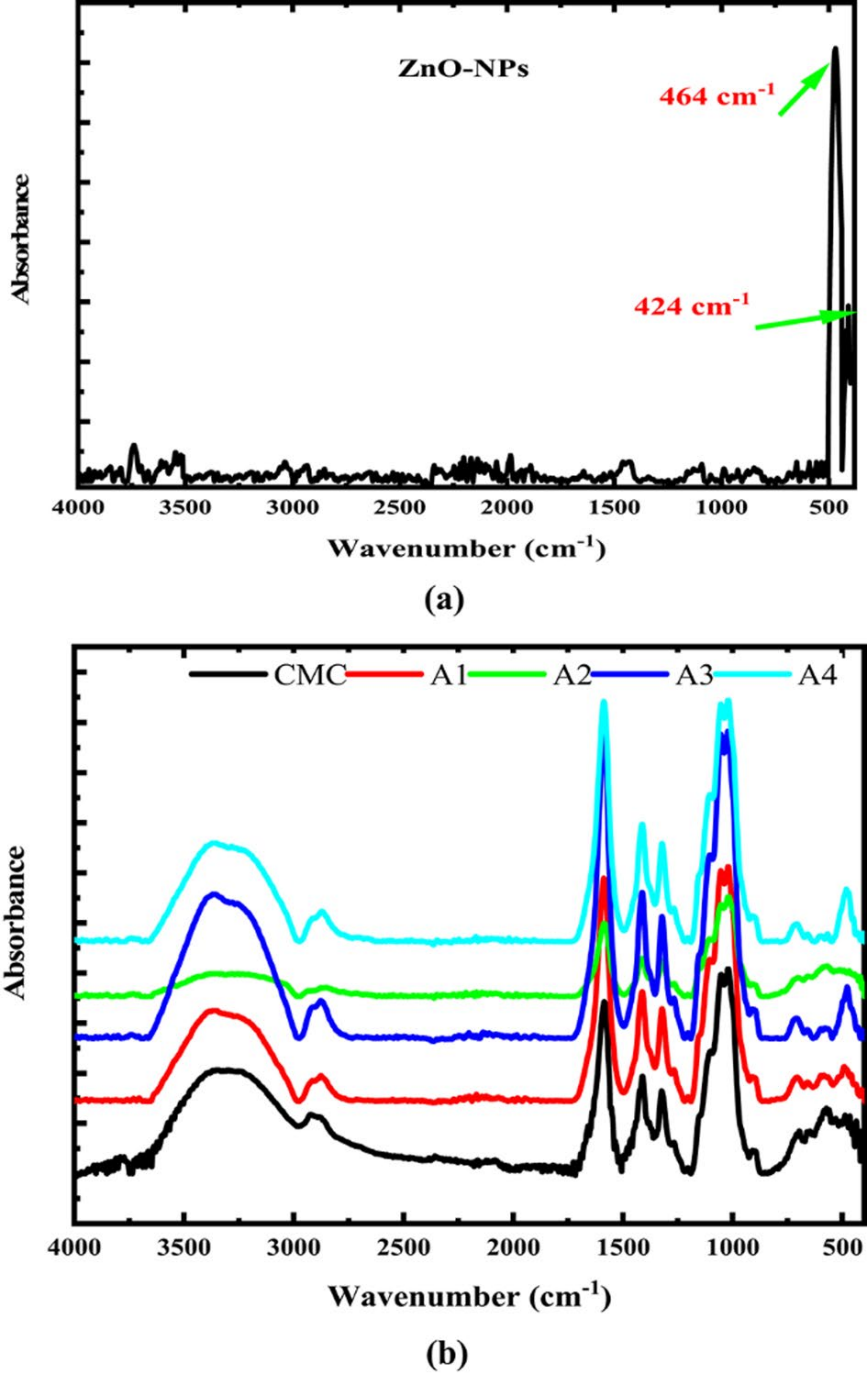
FTIR spectra of (**a**) ZnO-NPs and (**b**) pure CMC and CMC doped with 2, 4, 6, and 8 wt% of ZnO-NPs.

FTIR spectrophotometer was utilized to analyze the functional groups and chemical bonds of CMC/ZnO nanocomposite samples. Figure 1a illustrates the FTIR absorption spectrum of ZnO-NPs calcin ed at 500 °C. FTIR spectra investigated the formation of ZnO-NPs. As presented in the figure, the sharp absorption bands at 424 and 464 cm^−1^ were attributed to the Zn–O vibration mode^26^. The appearance of the 464 cm^−1^ absorption band confirmed that all Zn (OH)_2_ was transferred to ZnO. Additionally, the absence of absorption bands in the region extended from 4000 to 1500 cm^−1^ confirmed the purity of the synthesized ZnO-NPs and that the time of calcination and temperature are suitable to obtain ZnO-NPs.  

Figure 1b displays the FTIR spectra of pure CMC film recorded in the region of 4000–400 cm^–1^, with band allocations detailed in Table 2 according to prior research^25,30,31^. The extensive absorption band at 3267 cm^–1^ is ascribed to the O–H stretching vibration, whereas the band at 2881 cm^–1^ pertains to the asymmetric stretching of CH₂ groups. Distinct absorption bands at 1585 and 1412 cm^–1^ are attributed to the asymmetric stretching of the carboxyl group (COO^−^) and CH₂ scissoring, respectively. The bands at 1322 and 1270 cm^–1^ result from O–H bending and C–O–C vibrations. The bands at 1014 and 906 cm^–1^ correspond to C–O stretching of CH₂OCH₂ and CH₂ rocking vibrations, respectively. Supplementary bands within the range of 657–534 cm^–1^ correspond to the stretching and deformation of α-d-(1→4) and α-d-(1→6) links^32^.

Similar FTIR spectra can be seen in the CMC/ZnO nanocomposite films, coupled with typical ZnO bands. The inclusion of ZnO-NPs causes shifts in the CMC characteristic bands, indicating interactions between the CMC matrix and ZnO-NPs. The film with 2 wt% ZnO (sample A1) shifted the O–H stretching band to 3288 cm^–1^, whereas the COO−, O–H, and C–H stretching bands at 1585, 1413, and 1321 cm^–1^ were mostly unchanged. C–O stretching and CH₂ rocking vibrations were detected at 910 cm^–1^. A new band at 424 cm^–1^ shows the presence of Zn–O bonds, reflecting the complexation between CMC and ZnO-NPs.

By increasing the ZnO concentration to 4 wt% (sample A2), the O–H stretching band at 3261 cm^–1^ migrated to 3274 cm^–1^, whereas the CH asymmetrical stretching band moved to 2892 cm^–1^. ZnO’s distinctive bands at 414 and 426 cm^–1^ indicate strong interactions with CMC’s hydroxyl groups.

For 6 wt% ZnO (sample A3), the O–H stretching band shifted to 3276 cm^–1^, whereas the CH asymmetrical stretching was at 2891 cm^–1^. The presence of symmetric and asymmetric Zn–O stretching vibrations at 422 and 410 cm^–1^ confirms the complexation between CMC functional groups and ZnO-NPs. At the maximum ZnO concentration of 8 wt% (sample A4), the O–H stretching band changed to 3188 cm^–1^, whereas other functional groups exhibited small alterations. Overall, the strength of CMC bands increased as ZnO content rose, indicating that the polymer matrix and ZnO-NPs interacted strongly.

In the FTIR spectrum of all CMC nanocomposite samples, the noticeable increase in the band intensity around 3000–3500 cm^–1^ can be attributed to enhanced O–H stretching vibrations resulting from stronger interactions between the CMC matrix and ZnO-NPs. This may occurred due to two reasons which are: (1) the formation of new or stronger hydrogen bonds between the hydroxyl groups of CMC and the surface of ZnO-NPs, (2) additional hydroxyl groups were introduced to the CMC surface due to the addition of ZnO-NPs, and (3) enhanced interfacial interaction, where ZnO potentially acts as a coordination center, increasing dipole moments and intensifying the stretching vibration. Additionally, several studies show that incorporating ZnO into biopolymer matrices often increases the intensity or shifts the position of the broad O–H band due to hydrogen bonding or coordination interactions with surface Zn^2+^ sites. These strengthened interfacial interactions contribute to the improved structural integrity and optical properties of the nanocomposites^33–35^.

**Table 2:** FTIR band assignments of pristine CMC and CMC/ZnO nanocomposite samples containing 2, 4, 6, and 8 wt% ZnO-NPs.

Band position (cm^−1^)					Band assignment
CMC	A1	A2	A3	A4	
3261	3288	3274	3276	3188	Stretching of OH group
2919	2892	2892	2891	2619	C–H asymmetric stretching
1585	1585	1583	1583	1581	Carboxylate group (COO^−^) stretching
1412	1411	1411	1411	1415	CH_2_ scissoring
1322	1321	1319	1319	1317	OH bending
1052	1051	1053	1053	1051	C–O–C bending
1019	1018	1018	1018	1014	C–O bond of the CH_2_ OH group
912	910	912	919	919	C–O stretching + CH_2_ rocking motion
657−534	663−551	655−509	665−551	665−570	Ring deformation and stretching
–	424	426	422	428	Zn–O
–	–	414	410	408	Zn–O

### XRD results

**Fig. 2:**
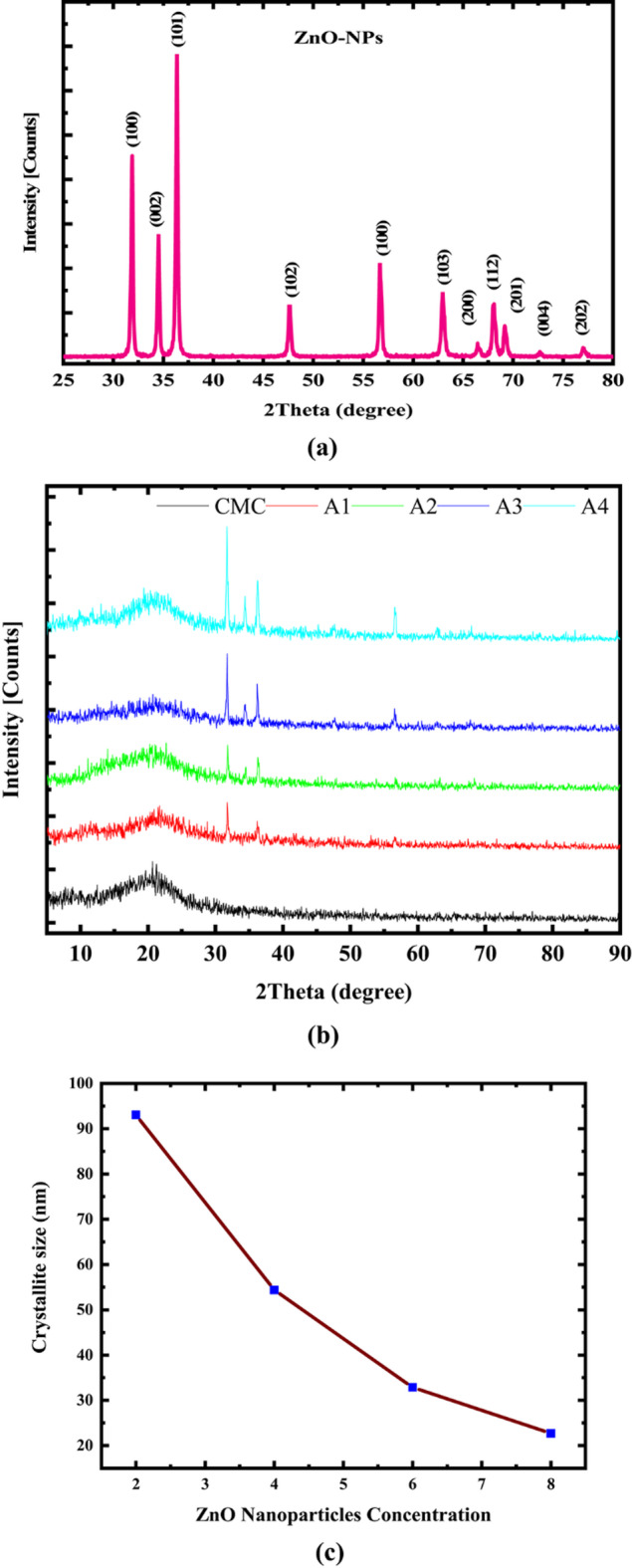
XRD patterns of: (**a**) ZnO-NPs and (**b**) CMC doped with 2, 4, 6, and 8 wt% of ZnO-NPs and (**c**) variation of crystallite size with ZnO concentration using Williamson–Hall method.

Figure 2 shows the XRD patterns of films made of pure CMC and CMC with varying amounts of ZnO-NPs. The XRD spectrum of ZnO-NPs synthesized by the precipitation method is presented in Fig. 2a. All the detectable peaks can be assigned to the ZnO wurtzite structure (reference code no: 01–082-9745), indicating the absence of secondary phases that typically appear when precursor residues remain. These results demonestrate the purity of the prepared ZnO-NPs and further confirm the FTIR findings. The spectrum clearly confirmed the crystalline nature of the prepared ZnO-NPs. The obtained XRD peaks at 31.84°, 34.49°, 36.33°, 47.61°, 56.67°, 62.93°, 66.45°, 68.02°, 69.16°, 72.63°, and 77.01° are indexed as: (100), (002), (101), (102), (110), (103), (200), (112), and (201), respectively, as presented in Fig. 2a. The crystallite size of ZnO-NPs was calculated for (100) plane, which indexed to the most prominent peak, and equals 39.51 nm (see Table 3). Consistent with earlier findings^25,27^, the absence of prominent diffraction peaks in the pure CMC film indicates that it is entirely amorphous. Extra diffraction peaks indicate that ZnO-NPs have been integrated into the CMC matrix. The 2θ values of 31.78°, 36.29°, 56.70°, 63.25°, and 68.36° that correspond to the (100), (101), (110), (103), and (201) crystal planes, reported in Table 3, reflects the presence of ZnO-NPs as presented in Fig. 2b. The 2θ values of 31.74°, 36.21°, 46.71°, 53.26°, and 56.56° correspond to the crystal planes (100), (101), (102), (003), and (110), respectively, in the XRD patterns of CMC/ZnO nanocomposite films^33^ as presented in Fig. 2b. Optimal crystallinity of the nanocomposite films is indicated by the fact that the ZnO diffraction peaks get more intense as the ZnO concentration increases. In nanocomposites, the CMC peak intensity drops as ZnO-NPs concentration raises (see Fig. 2b), compared to the pure CMC. This could be because the average crystallite size is reduced as a result of interactions between ZnO-NPs and the CMC matrix, which minimize agglomeration^26,36^.

In samples with 6 and 8 wt% ZnO, extra diffraction peaks are observed: at 34.37° and 47.57° for the (002) and (102) planes in the 6 wt% ZnO-NPs sample, while for the 8 wt% ZnO-NPs sample, peaks are present at 34.37°, 47.57°, and 81.46° corresponding to the (002), (102), and (104) planes. The observed peak shifts suggest structural changes in CMC caused by ZnO-NPs.

The crystallite size (D), microstrain (ε), dislocation density (δ), and the number of crystallites (Nc) for the dominant (100) peak were determined via the full width at half maximum (FWHM) of the diffraction peaks, as presented in Table 3. The mean crystallite size was calculated employing Debye–Scherrer’s equation:

1 $$\boldsymbol{D}=\frac{\boldsymbol{K}\boldsymbol{\lambda}}{\boldsymbol{\beta}\boldsymbol{C}\boldsymbol{o}\boldsymbol{s}\boldsymbol{\theta}}$$

where β is the FWHM, λ = 0.154 nm is the Cu K_α_ wavelength, θ is the Bragg angle, and D is the crystallite size in nm. Microstrain (ε), dislocation density (δ), and the number of crystallites (Nc) were calculated using:

2 $$\boldsymbol{\varepsilon}=\frac{\boldsymbol{\beta}\boldsymbol{c}\boldsymbol{o}\boldsymbol{s}\boldsymbol{\theta}}{{\mathbf{4}}}$$

3 $$\boldsymbol{\delta}=\frac{1}{{\boldsymbol{D}}^{{\mathbf{2}}}}$$

4 $${\boldsymbol{N}}_{\boldsymbol{c}}=\frac{\boldsymbol{t}}{{\boldsymbol{D}}^{{\mathbf{3}}}}$$

5 $$\boldsymbol{\beta}\boldsymbol{c}\boldsymbol{o}\boldsymbol{s}\boldsymbol{\theta}=\frac{\boldsymbol{K}\boldsymbol{\lambda}}{\boldsymbol{D}}+{\mathbf{4}} \boldsymbol{\varepsilon}\boldsymbol{s}\boldsymbol{i}\boldsymbol{n}\boldsymbol{\theta}$$

where t, represents the thickness of the film. The findings indicate that the size of the crystallites diminishes as the ZnO content rises, achieving 32.47 nm for the sample with 8 wt% ZnO-NPs as presented in Fig. 2c. This decrease is ascribed to lattice distortion and internal stress caused by ZnO-NPs^37^. Moreover, the reduction in peak intensity relative to pure CMC suggests a rise in defect density. Micro-strain and dislocation density also rise with increased ZnO content, as detailed in Table 2. The observed increase in the dislocation density of CMC/ZnO nanocomposites with increasing ZnO-NPs concentration revealed that the nanocomposite samples had a low degree of crystallinity.

Additionally, the crystallite size was calculated using both the Scherrer method and the Williamson–Hall (W–H) method, and noticeable differences were observed between the two sets of values. The Scherrer equation assumes that the broadening of XRD peaks arises solely from crystallite size and does not account for any contribution from lattice strain or structural defects, which often leads to simplified or overestimated crystallite size values. In contrast, the Williamson–Hall approach separates the effects of crystallite size and microstrain on peak broadening, providing more realistic values when the material contains internal strain or lattice imperfections. Several nanomaterials, such as ZnO, are known to exhibit compressive strain due to lattice mismatch or internal defects, which directly influences the XRD peak broadening and the derived structural parameters^38^.

Based on this understanding, the samples in which the W–H crystallite size (D) was smaller than the Scherrer value, indicate the presence of compressive strain or structural defects that increase peak broadening. This effect cannot explain using the Scherrer method. The unusually larger W–H crystallite size observed for sample A1 can be attributed to its very low nanofiller loading relative to the polymer matrix. Low filler content typically reduces lattice defects and microstrain at the polymer–filler interface, leading to minimal strain-induced XRD broadening. Since the Williamson–Hall method incorporates the effect of microstrain, weak strain broadening results in a larger calculated crystallite size, consistent with previous findings showing that reduced defect density lowers microstrain contributions in nanocomposites^39^.

**Table 3:** Crystallite Size (D), dislocation density (δ), micro strain (ε) and number of crystallites (Nc) of the most prominent peak of ZnO-NPs [i.e., plane (100)] in all CMC/ZnO nanocomposite films.

Sample	Sherrer method				Williamson-Hall method
	D (nm)	δ (nm^−2^) × 10^−3^	ε × 10^−3^	*N*_*c*_ × 10^−2^	D (nm)
ZnO	39.51	0.64	2.82	16200.16	35.37
A1	103.24	0.09	1.23	3.27	93.06
A2	41.61	0.58	3.04	38.86	54.37
A3	40.07	0.62	3.17	40.42	32.86
A4	32.47	0.95	3.91	67.16	22.69

### Optical results

#### Determination of absorption parameters

Optical absorption spectra are a great tool for studying the energy band gap and band structure of crystalline and amorphous materials. The absorption spectrum shows electronic transitions in the higher-energy region and atomic vibrations in the lower-energy region. Researchers can learn more about a material’s electrical structure and optical constants by analyzing its UV-Vis optical characteristics^40,42,42^. The creation of photo-stable, photo-resistant, and photo-degradable polymers relies heavily on understanding their photo-stability and degradation mechanisms. This is because polymers are extensively employed under the sunlight^42,43^. In this context, the fundamental optical properties of CMC/ZnO nanocomposites, including absorbance, transmittances, and reflectance, were investigated.

**Fig. 3:**
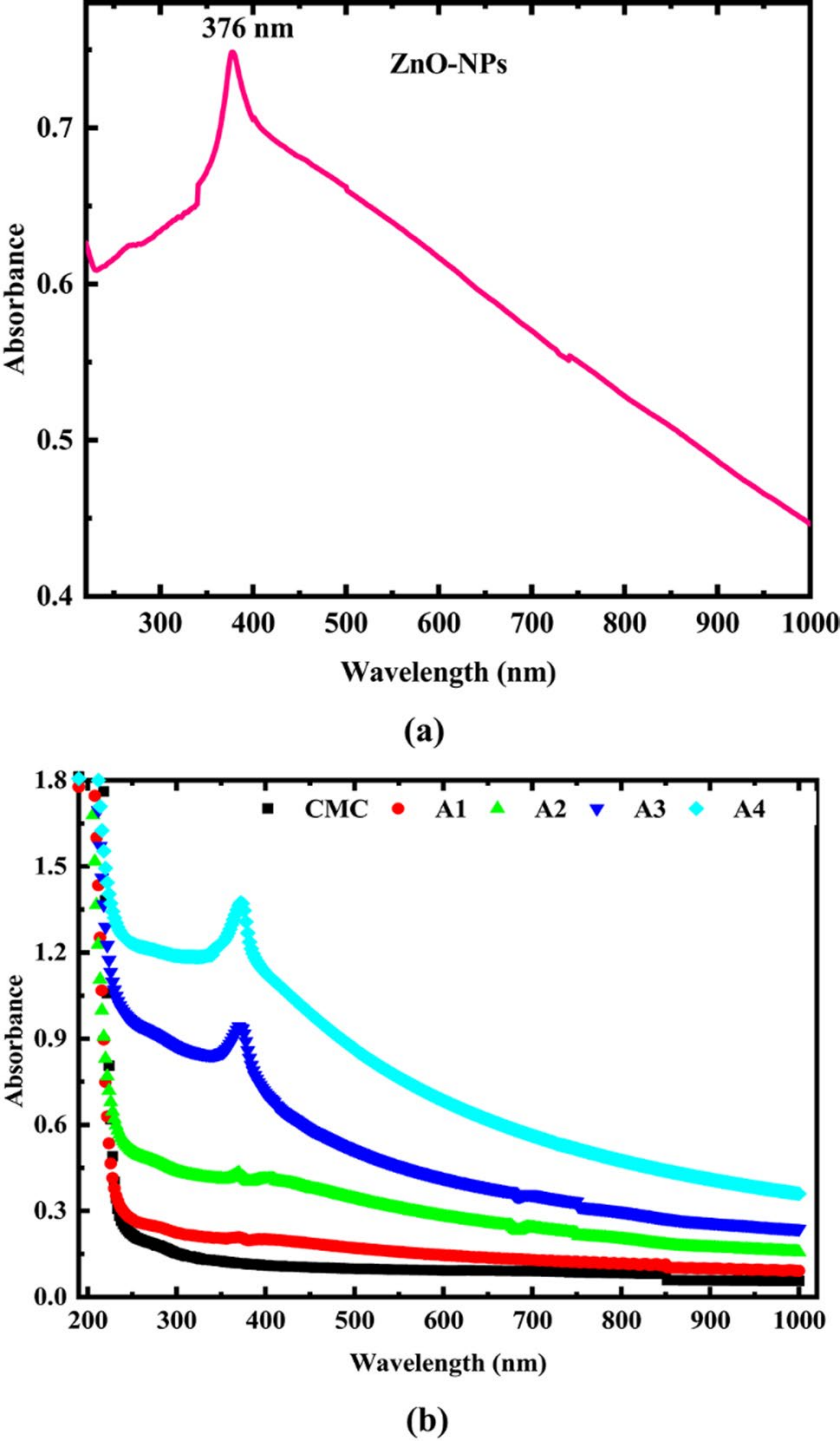
UV-Vis absorbance spectra of: (**a**) ZnO-NPs and (**b**) pure CMC and CMC doped with 2, 4, 6, and 8 wt% of ZnO-NPs.

The optical absorption spectra of ZnO-NPs, pure CMC, and CMC/ZnO nanocomposite films with varying ZnO-NPs concentrations (2, 4, 6, and 8 wt%) are displayed in Fig. 3. Absorption spectra of ZnO-NPs exhibit a strong absorption peak at about 376 nm due to bandgap absorption from removed electrons to the conduction band. Furthermore, the sharp absorption peak observed confirmed the narrow particle size distribution of the prepared ZnO-NPs. Thus, ZnO-NPs absorb well in the UV region (200–400) nm, making them suitable for optical applications such as filters and sunscreen protectors. When ZnO-NPs are added, the absorbance of pure CMC goes up a lot, and the absorption edge moves toward longer wavelengths. The absorption intensity rises as the ZnO content rises, which shows that the CMC matrix and ZnO-NPs interact strongly with each other. At 376 nm, the ZnO-NPs absorption band appears, and it moves a little to longer wavelengths as the concentration of nanoparticles increases. Changes in the distribution of particle sizes are what cause the differences in extinction band intensities that we see^44,45^.

#### Reflectance analysis

**Fig. 4:**
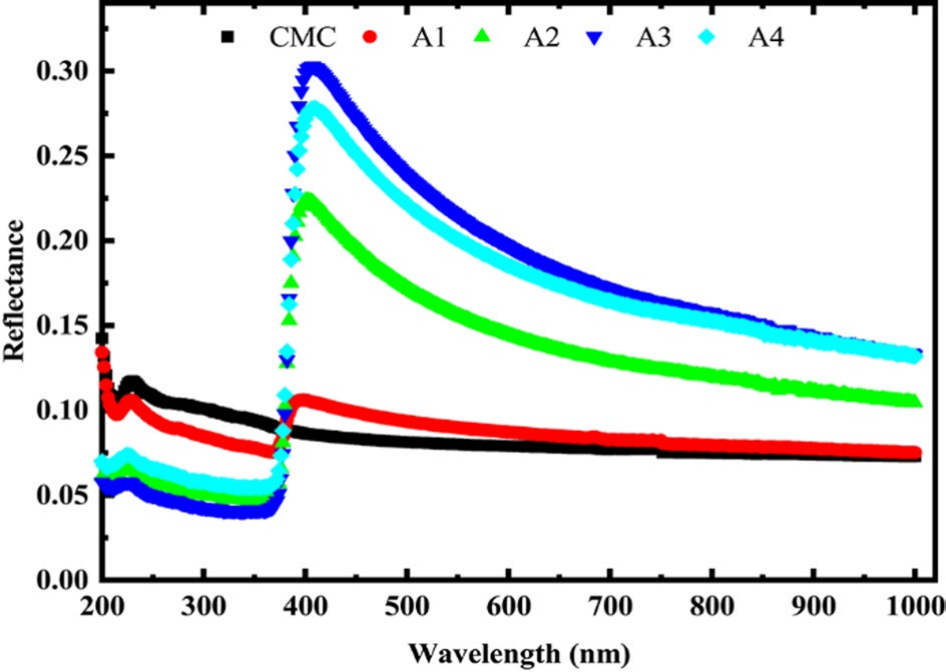
Reflectance spectra of CMC and the CMC doped with different concentrations of ZnO.

As illustrated in Fig. 4, the reflectance spectra, R(λ), of both pure CMC and CMC/ZnO nanocomposite films with varying ZnO-NPs concentrations (2, 4, 6, and 8 wt%) were obtained within the 200–1000 nm wavelength range. As the ZnO-NPs content increases, the reflectance of pure CMC drops in the UV range (200–400 nm), suggesting that UV light absorption is the main process.

When the wavelength is increased from 400 to 600 nm, the reflectance of all samples drops dramatically. The reflectance of pure CMC gradually drops between 400 and 1000 nm and continuously drops between 200 and 400 nm. As a result of the strong interaction between the polymer matrix and the ZnO-NPs, CMC/ZnO nanocomposite films fail to achieve saturation within the measured spectral range. The changes in reflectance that were seen when ZnO was added may be due to changes in the packing density of CMC^46^. Accordingly, CMC/ZnO nanocomposite films are good candidates for use in marking paints, reflective tapes, leather, textiles, and safety fabrics because they reflect a lot of light and block UV rays.

#### Absorption coefficient

**Fig. 5:**
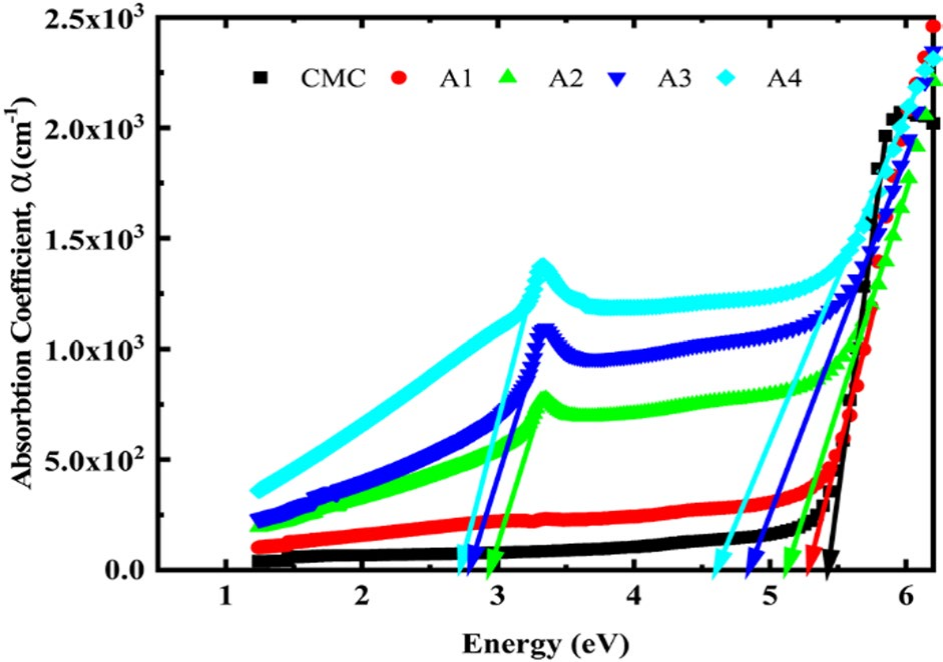
Absorption coefficient (α) of CMC and the CMC doped with different concentrations of ZnO.

How far light of a specific wavelength may penetrate into a substance before being absorbed is indicated by the absorption coefficient (α)^27^. Absorption coefficient was calculated using the following relation:

6 $${\boldsymbol{\upalpha }} = \frac{{\mathbf{1}}}{\boldsymbol{t}}{\mathbf{ln}}\left[ {\left( {\frac{{\left( {{\mathbf{1}} - \boldsymbol{R}} \right)^{{\mathbf{2}}} }}{{{\mathbf{2}}\boldsymbol{T}}}} \right) + \left( {\frac{{\left( {{\mathbf{1}} - \boldsymbol{R}} \right)^{{\mathbf{4}}} }}{{{\mathbf{4}}\boldsymbol{T}^{{\mathbf{2}}} }} + \boldsymbol{R}^{{\mathbf{2}}} } \right)^{{{\mathbf{1}}/{\mathbf{2}}}} } \right]$$

where t, is the film thickness, R, is the reflectance, and T is the transmittance. Figure 5 displays the relationship between photon energy (hv) and the absorption coefficient of the prepared nanocomposite films under study. As the concentration of ZnO rises, the absorption coefficient also rises dramatically. The absorption spectra show that pure CMC has an absorption edge. The red shift occurs when the concentration of ZnO increases and the absorption edge moves toward lower energies. Nanocomposite films also show a second absorption edge, which moves to lower energy as the ZnO concentration increases. The red shift is due to n–π* electronic transitions, indicating increased interatomic interactions and a reduction in the optical energy gap with rising ZnO concentration. The increased absorption in CMC/ZnO nanocomposite films is linked to charge transfer from the valence band, predominantly constituted by O 2p orbitals in the metal oxide, to the conduction band comprised of Zn 3d orbitals^43,44^. The absorption characteristics of pure CMC align with previously documented findings^25,27,28,30^.

#### Optical band gap determination

**Fig. 6:**
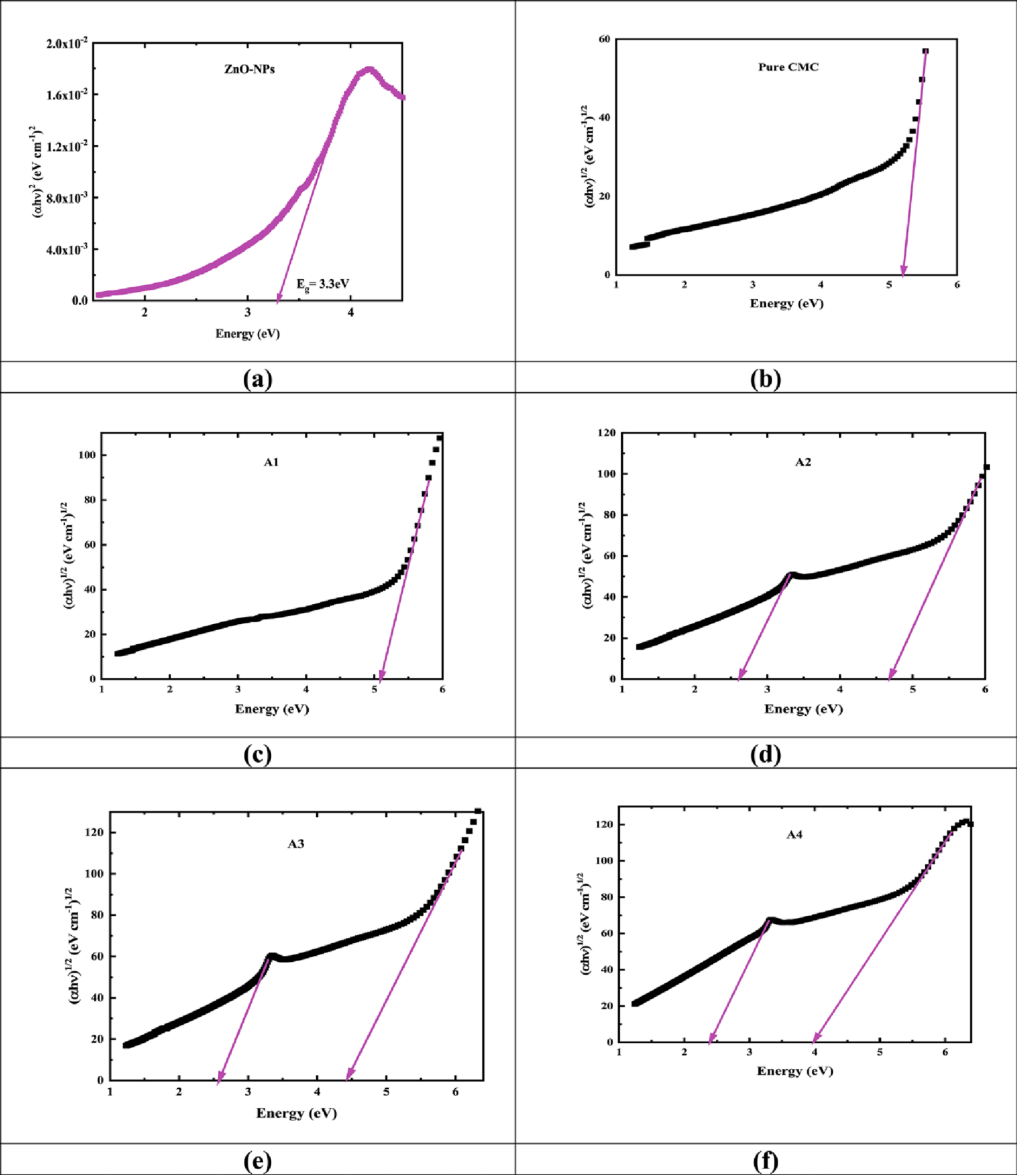

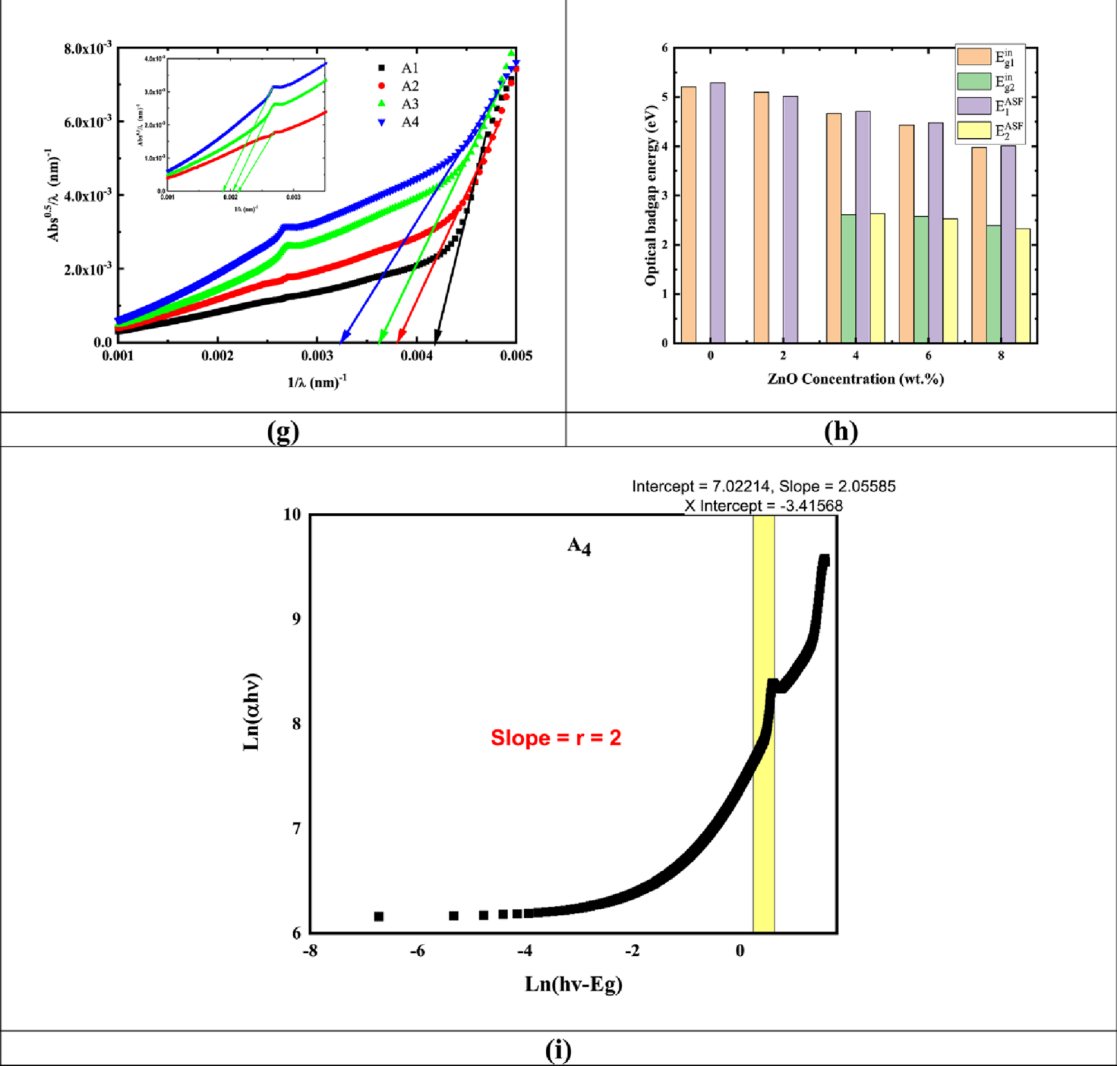
Tauc plots (αhν)^1/2^ as a function of photon energy (hν) of: (**a**) ZnO-NPs, (**b**) pure CMC, (**c**) CMC/2wt.% ZnO, (**d**) CMC/4wt.% ZnO, (**e**) CMC/6wt.% ZnO, (**f**) CMC/8wt.% ZnO; (**g**) Absorption Spectrum Fitting (ASF) plot (Abs)^1/2^/λ as a function of 1/λ, (**h**) Comparison between Tauc and ASF models for energy gap verification, and (**i**) variation of ln(αhν) versus ln(hν − E_g_) for sample A4.

The optical bandgap energy (Eg) of the CMC/ZnO nanocomposite films is a critical characteristic for assessing their prospective uses. The absorption coefficient can assess the nature of electronic transitions and the optical bandgap through the established Tauc relation:

7 $$\left( {{\boldsymbol{\upalpha}} {\mathbf{h}}{\boldsymbol{\upnu }}} \right) = {\mathbf{B}}\left( {{\mathbf{h}} {\boldsymbol{\upnu }} - {\mathbf{E}}_{{\mathbf{g}}} } \right)^{{\mathbf{r}}}$$

where B represents a constant, hv denotes the photon energy, and r reflects the type of transition. The Tauc method is widely applied for estimating the optical band gap of semiconductor materials, including both bulk solids and thin films, and remains a standard tool for analyzing UV–Vis absorption spectra in thin-film systems. Although the method was originally developed for amorphous semiconductors, it has since been routinely adapted to various classes of polycrystalline and thin-film materials due to its simplicity and its ability to provide a reliable estimate of the absorption edge when appropriate assumptions are met. Pure CMC is noted to possess an indirect optical bandgap as discussed previously^25,49^; hence, *r* = 1/2. The optical bandgap (E_g_) of and all nanocomposites was obtained by graphing (αhν)^1/2^ against hν, as seen in Fig. 6b: g. Table 4 summarizes the E_g_ values for pure CMC and CMC doped with 2, 4, 6, and 8 wt% of ZnO-NPs. The optical transitions were determined by projecting the linear segment of the Tauc figure to the energy axis, where (αhν)^1/r^=0. All nanocomposite specimens have two optical transitions. The lower-energy transition pertains to the n–π* transition (Onset gap), which results from the alternation of electron-rich and electron-deficient units throughout the polymer chain. The higher-energy transition pertains to the π–π* transition, linked to the HOMO–LUMO gap.

For ZnO-NPs, by plotting (αhν)^2^ as a function of energy, the value of the optical bandgap was obtained to be ~ 3.3 eV as presented in Fig. 6a. The strong absorption in the UV region demonstrated that the synthesized ZnO-NPs have a good capacity for UV-protection applications. To confirm the optical bandgap value of ZnO-NPs obtained in this work, it can be compared with previously reported ZnO and ZnO-based nanostructures. Undoped ZnO typically exhibits a wide band gap around 3.37 eV as reported in earlier literature^47^. Several studies show that synthesis conditions can significantly lower this value, reaching ≈ 2.70 eV for ZnO prepared at low calcination temperatures^48,49^. Doping and nanocomposite formation can either reduce or widen the band gap: for example, Sr-doped ZnO decreased from 3.16 to 2.90 eV^50^, Ni-doped ZnO decreased from 3.37 to 2.90 eV^51^, while MgZnO nanocomposites exhibited slightly higher band-gap values between 3.47 and 3.51 eV^52^.

Pure CMC possesses an indirect bandgap of 5.21 eV as reported previously^25,27,28^. The integration of ZnO-NPs diminishes the optical bandgap, resulting in a reduction of the HOMO–LUMO gap from 5.21 to 3.98 eV, while the Onset gap decreases to 2.39 eV at the maximum ZnO concentration. The decrease in optical bandgap can be elucidated by the density of states hypothesis put out by Mott and Davis^53,54^.

The observed band-gap narrowing can be attributed to the formation of localized electronic states generated by structural disorder and defect-related potential fluctuations. Structural distortions such as variations in bond lengths, bond angles, and local strain fields create fluctuations at the band edges, producing exponential Urbach tail states that effectively reduce the optical bandgap^55^. Defect states arising from vacancies and impurity-related disorder further deepen these localized tail states and broaden the band-edge region, thereby enhancing the narrowing effect^56^. Electrostatic disorder at interfaces may also contribute to the tail-state density and thus to the apparent band-gap reduction. Together, these mechanisms explain the intrinsic link between disorder-induced localized states and the observed band-gap narrowing in the material^57^. The bandgap values of CMC/ZnO nanocomposites fall well within the expected ZnO window, indicating that the reduction in band gap due to nanoparticle incorporation is consistent with previously observed trends of defect formation, polymer–oxide interaction, or particle size effects.

The UV absorption spectra of the nanocomposites were subjected to the Absorption Spectrum Fitting (ASF) model in order to provide additional validation to the optical bandgap values that were obtained using the Tauc method^58^. By using absorbance data alone, the ASF model may calculate the optical gap energy (${E}_{Opt}^{ASF}$) without knowing the film thickness. The ASF method determines the optical gap energy as:

8 $${\boldsymbol{E}}_{\boldsymbol{O}\boldsymbol{p}\boldsymbol{t}}^{\boldsymbol{A}\boldsymbol{S}\boldsymbol{F}}=\frac{{\mathbf{1240}}}{{\boldsymbol{\lambda}}_{\boldsymbol{O}\boldsymbol{p}\boldsymbol{t}}^{\boldsymbol{A}\boldsymbol{S}\boldsymbol{F}}}$$

where ${\boldsymbol{\lambda}}_{\boldsymbol{O}\boldsymbol{p}\boldsymbol{t}}^{\boldsymbol{A}\boldsymbol{S}\boldsymbol{F}}$, is the wavelength corresponding to the absorption onset. Figure 6g displays the change in of (Abs)^1/2^/λ relative to 1/λ for the nanocomposite films of CMC and ZnO. Table 4 summarizes the optical bandgap values which are obtained by extrapolating the linear part of each curve.

Figure 6g shows that CMC displays a single transition (HOMO/LUMO bandgap); however, increasing the ZnO-NPs concentration to 4, 6, and 8 wt% results in the appearance of an additional transition associated with the onset gap. The HOMO/LUMO bandgap obtained using ASF model of CMC is 5.29 eV as presented in Table 4. This value decreased to 5.02, 4.71, 4.48, and 4.01 eV due to the addition of 2, 4, 6, and 8wt.% of ZnO-NPs, respectively. Meanwhile, the onset gap decreased to 2.63, 2.53, and 2.33 for CMC doped with 4, 6, and 8wt.% of ZnO-NPs, respectively. A quantitative assessment of the optical bandgap obtained using the ASF method shows excellent consistency with the values derived from the Tauc plot (see Fig. 6h). The absolute differences between both methods were found to be very small (0.02–0.08 eV for the highest transitions and 0.02–0.06 eV for the low transition), yielding an average deviation of approximately 0.04 eV. This close agreement confirms the reliability and validity of the ASF method for determining the optical bandgap of CMC/ZnO polymer nanocomposites.

Additionally, the indirect transition model was validated for both pristine CMC and ZnO-containing nanocomposites by examining the shape of the absorption edge and the linearity of the corresponding Tauc plots. Moreover, incorporation of ZnO-NPs modified the bandgap value but did not alter the characteristic linear behavior associated with an indirect allowed transition (see Fig. 6). Direct-transition fitting did not yield comparable linearity, supporting that the transition mechanism remains indirect after nanocomposite formation.

In order to investigate the absorption-edge behavior, the logarithmic form of the Bain–Oppenheimer relation was employed:

9 $$\frac{\boldsymbol{d}\left[\boldsymbol{l}\boldsymbol{n} \left(\boldsymbol{\alpha}\boldsymbol{h}\boldsymbol{\upsilon}\right)\right]}{\boldsymbol{d}\left(\boldsymbol{h}\boldsymbol{\upsilon}\right)}=\frac{\boldsymbol{r}}{\left(\boldsymbol{h}\boldsymbol{\upsilon}-{\boldsymbol{E}}_{\boldsymbol{g}}\right)}$$

This relation enables determining the electronic transition type through the exponent *r*. To extract this value for sample A4, the relationship between ln(αhν) and ln(hν − E_g_) was plotted in the spectral region just above the band edge. The linear portion of the plot exhibited a clear straight-line behavior, allowing accurate determination of the slope.

From the fitted linear region, the calculated slope was found to be *r* = 2, a value characteristic of an indirect allowed transition, according to the established classification of optical transition exponents reported in previous studies that applied the same analytical approach^59^. This confirms that the investigated material exhibits an indirect optical transition, consistent with the expected behavior of semiconducting systems where the momentum-dependent transition appears with a slope close to 2 in the ln(αhν) versus ln(hν − E_g_) representation.

**Table 4:** Indirect allowed optical bandgap energies determined using Tauc and ASF models, and Urbach energy (E_U_) for pure CMC and CMC/ZnO nanocomposite films containing 2, 4, 6, and 8 wt% ZnO-NPs.

CMC/ZnO-NPs	$E_{g1}^{in}$ (eV)	$E_{g2}^{in}$ (eV)	$E_{Opt1}^{ASF}$ (eV)	$E_{Opt2}^{ASF}$ (eV)	E_U_ (eV)
0	5.21	–	5.29	–	0.21
2	5.10	–	5.02	–	0.31
4	4.67	2.61	4.71	2.63	0.71
6	4.43	2.58	4.48	2.53	0.91
8	3.98	2.39	4.01	2.33	1.11

#### Urbach energy

The absorption edge provides useful information regarding structural disorder for amorphous materials. The exponential absorption tail arises when short-range structural distortions, bond-angle/length fluctuations, and lattice strains generate localized states that extend into the forbidden band region^60^. Defect centers such as vacancies, dislocations, impurity atoms, and dopant-induced defect clusters further broaden these tail states and increase E_U_ by enhancing the density of localized electronic states near the band edges^61,62^.

**Fig. 7:**
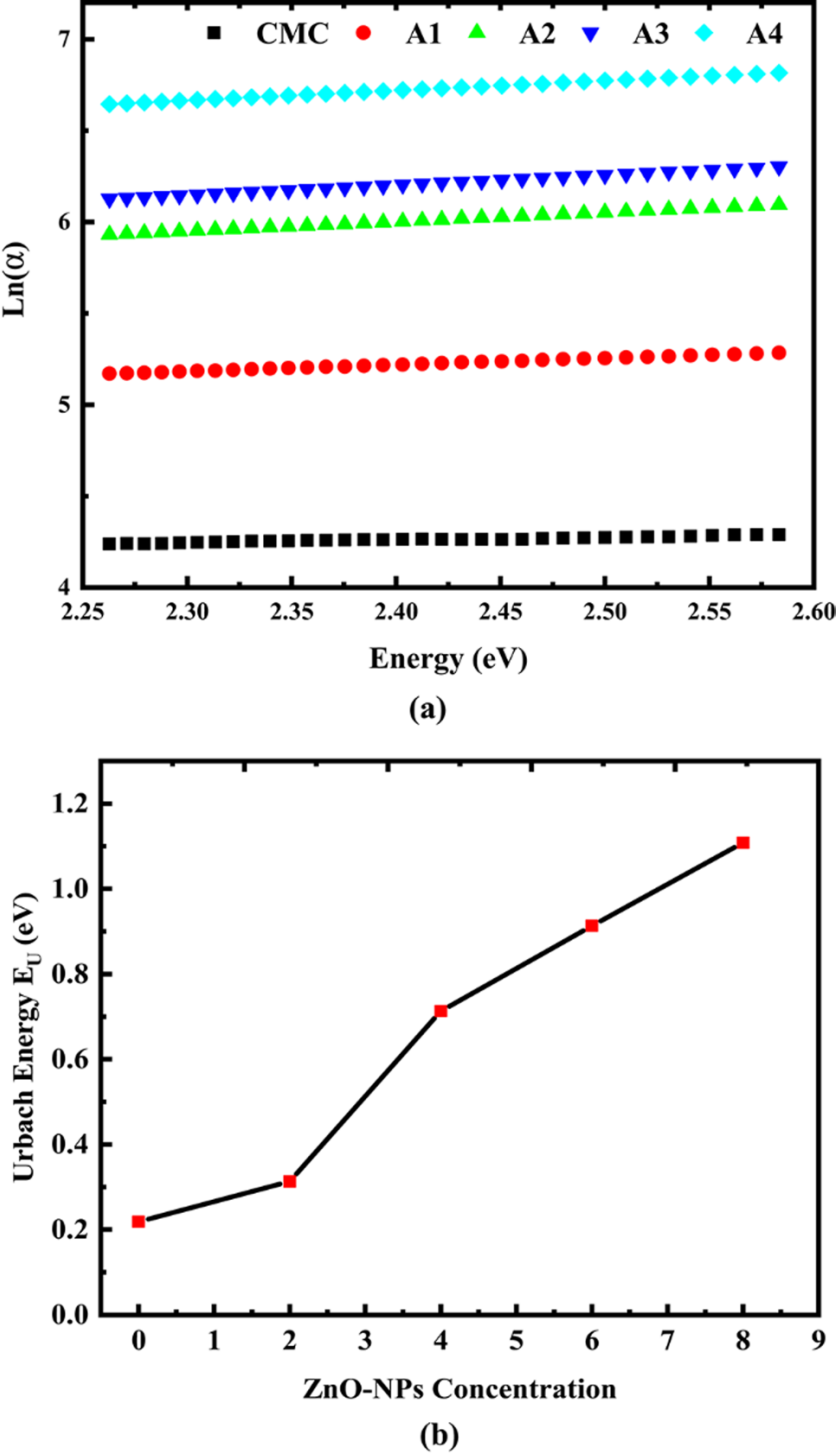
(**a**) ln(α) versus photon energy (hν) of CMC and CMC doped with 2, 4, 6, and 8 wt% of ZnO-NPs and (**b**) Variation of Urbach energy (E_U_) as a function of ZnO-NPs concentration.

Additionally, electron–phonon interactions contribute dynamic disorder that further modulates tail-state broadening^63,64^. Therefore, an increase in Urbach energy directly reflects higher degrees of structural disorder, enhanced defect density, and phonon-assisted localization, whereas lower E_U_ indicates improved structural order and reduced tail-state formation. The Urbach energy (E_U_) can be determined using the empirical Urbach relation:

10 $$\boldsymbol{\alpha}={\boldsymbol{\alpha}}_{\boldsymbol{o}}\mathbf{exp} \left(\frac{\boldsymbol{h}\boldsymbol{\upsilon}}{{\boldsymbol{E}}_{\boldsymbol{U}}}\right)$$

where E_U,_ stands for the level of disorder in the material and αo, is a constant associated with the characteristics of the polymer. Figure 7a illustrates the correlation between ln(α) and photon energy (hv). The linear fits demonstrate that inter-band transitions in the CMC/ZnO nanocomposites obey the Urbach rule. Figure 7b illustrates the correlation between Urbach energy and ZnO-NPs concentration. The inverse of the slope of each line in Fig. 7a was utilized to compute E_U_ for all samples. The enhancement of E_U_ correlates with the rising concentration of ZnO-NPs, suggesting the emergence of supplementary localized states within the bandgap. The rise in disorder could originate from an elevated concentration of point defects, resulting in band-to-tail and tail-to-tail transitions^65^. These findings align with the structural study of the nanocomposites.

### Optical dispersion parameters

The term “refractive index” (n) refers to the amount of electromagnetic radiation that is able to penetrate an optical medium. The dispersion of the refractive index is an important element in studying the properties of optical materials such as filters. Additionally, it is an important factor in the analysis of spectrum dispersion and optical communication. The link between the energy bandgap and the value of n is a crucial component in the process of establishing the optical and electrical properties of semiconductors. Since materials that have high refractive indices and low bandgap energies often display greater performance in optical filters. The extinction coefficient, denoted by the letter k, is a measure of the amount of energy that is lost as electromagnetic radiation travels through a substance. Using the following relations^66^, it is possible to derive the refractive index (n) as well as the extinction coefficient (k) from the transmittance and reflectance that have been measured.

11 $$\boldsymbol{\upkappa}=\frac{\boldsymbol{\upalpha}\boldsymbol{\uplambda}}{{\mathbf{4}}\boldsymbol{\uppi}}$$

12 $${\mathbf{n}} = \left( {\frac{{\left( {{\mathbf{1}} + {\mathbf{R}}} \right)}}{{\left( {{\mathbf{1}} - {\mathbf{R}}} \right)}} + \sqrt {\left( {\frac{{{\mathbf{4}}{\mathbf{R}}}}{{\left( {{\mathbf{1}} - {\mathbf{R}}} \right)^{2} }}} \right) - {\mathbf{\kappa }}^{{\mathbf{2}}} } } \right)$$

**Fig. 8:**
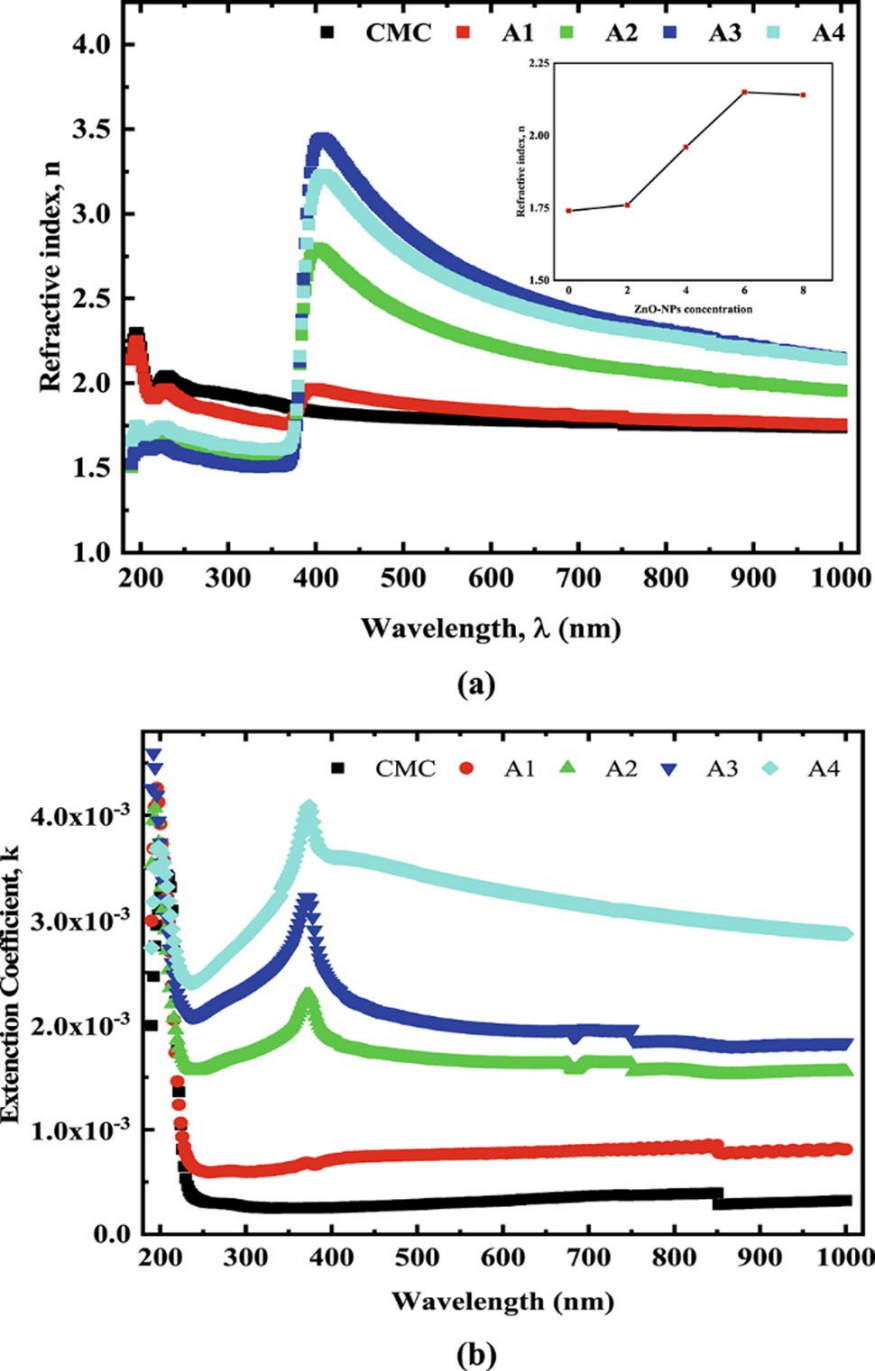
(**a**) Refractive index (n)   and (**b**) Extinction coefficient (k) spectra of CMC and CMC doped with 2, 4, 6, and 8 wt% of ZnO-NPs.

The spectrum distribution of the refractive index (n) is depicted in Fig. 8a for both pure CMC and CMC/ZnO nanocomposite films. A rapid fall in n is observed at shorter wavelengths, followed by the appearance of two peaks, which is indicative of anomalous dispersion. An observation of normal dispersion can be obtained at wavelengths that extend beyond the absorption region and into the near-infrared (NIR) spectral region. As presented in Table 5, the refractive index of pure CMC increased from 1.78 to 1.84, 2.23, 2.51, and 2.60 due to the addition of 2, 4, 6, and 8 wt% of ZnO-NPs. The observed increase in refractive index may be associated with a change in the film’s effective density that occurs with an increase in the concentration of ZnO-NPs. Additionally, the establishment of intermolecular hydrogen bonds between ZnO-NPs and CMC enhances film density, leading to elevated refractive indices, in accordance with the FTIR findings. Moreover, the refractive index exhibits a progressive decline with increasing wavelength in the region of 460–1000 nm prior to achieving saturation. This behavior, representative of standard dispersion, can be characterized using the Single-Oscillator model.

It is important to note that the variation observed in the measured refractive index of the CMC/ZnO thin films may not solely originate from compositional or structural differences within the material. In thin-film systems, surface roughness can also influence optical behavior by increasing light scattering or altering the effective optical path. Since atomic force microscope measurements were not conducted in this study, the possible contribution of surface roughness to the observed refractive-index variation cannot be fully evaluated. Therefore, this factor should be acknowledged as a limitation, as it may play a role in accurately interpreting the optical results.

Figure 8b illustrates the extinction coefficient (k) in relation to wavelength. With the increase in ZnO-NPs concentration, k likewise rises. In the 200–250 nm region, k is minimal owing to the great transparency of CMC films. In the 250–376 nm range, the extinction coefficient (k) exhibits a considerable rise for all nanocomposite films, attributable to the presence of ZnO-NPs. The significant rise in k is attributable to the inherent ultraviolet absorption characteristics of ZnO-NPs, which possesses a broad bandgap (~ 3.37 eV) and exhibits substantial absorption of ultraviolet light. The use of ZnO-NPs improves the optical absorption of the nanocomposite films, leading to elevated extinction coefficient values relative to pure CMC.

The optical oscillator parameters, high-frequency dielectric constant, and lattice dielectric constant were computed from the normal dispersion region utilizing the Single-Oscillator model established by Wemple and DiDomenico. The differences in the dispersion curves for pure CMC and CMC/ZnO nanocomposite films can be elucidated by the oscillator model.

The Single-Oscillator model correlates the refractive index (n) of the materials with their electronic structure through the following relation^67^:

13 $${\boldsymbol{n}}^{{\mathbf{2}}}-{\mathbf{1}}=\frac{{\boldsymbol{E}}_{\boldsymbol{o}}{\boldsymbol{E}}_{\boldsymbol{d}}}{\left({\boldsymbol{E}}_{\boldsymbol{o}}^{{\mathbf{2}}}-{\left(\boldsymbol{h}\boldsymbol{\upsilon}\right)}^{{\mathbf{2}}}\right)}$$

where E_o_ denotes the single-oscillator energy representing the average energy of all electronic excitations, and E_d_ signifies the dispersion energy, which is proportional to the average oscillator strength.

**Fig. 9:**
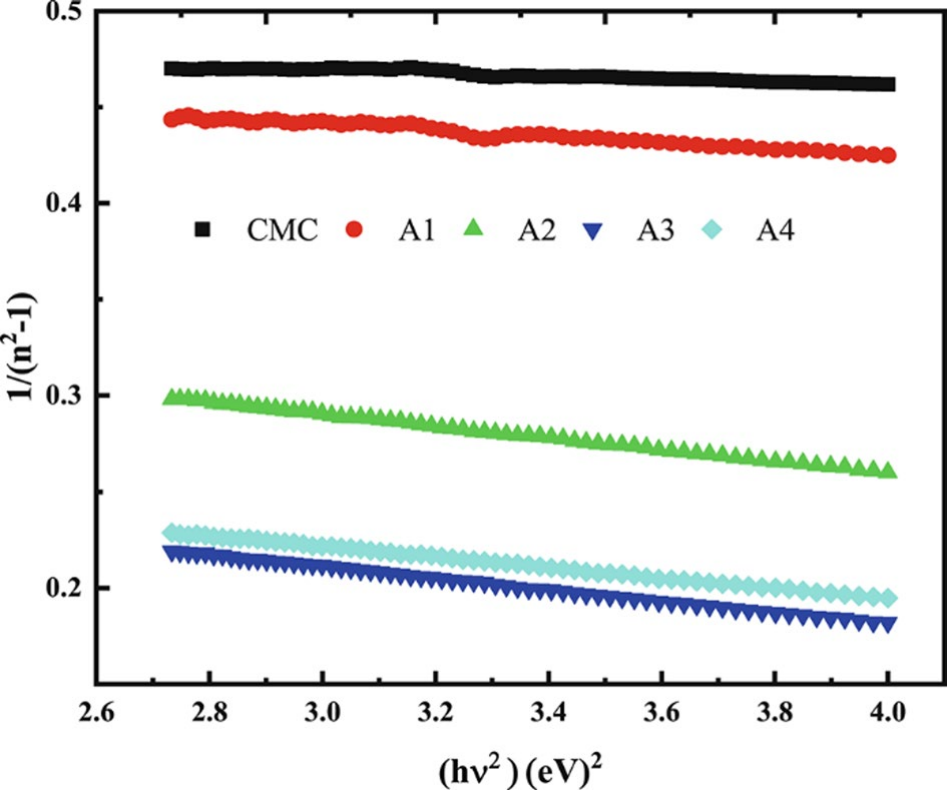
Plot of (n^2^−1)^−1^ versus (hν) of CMC and CMC doped with 2, 4, 6, and 8 wt% of ZnO-NPs. The slope and intercept of the linear region were used to calculate the Single-Oscillator parameters E_o_ and E_d_.

**Fig. 10:**
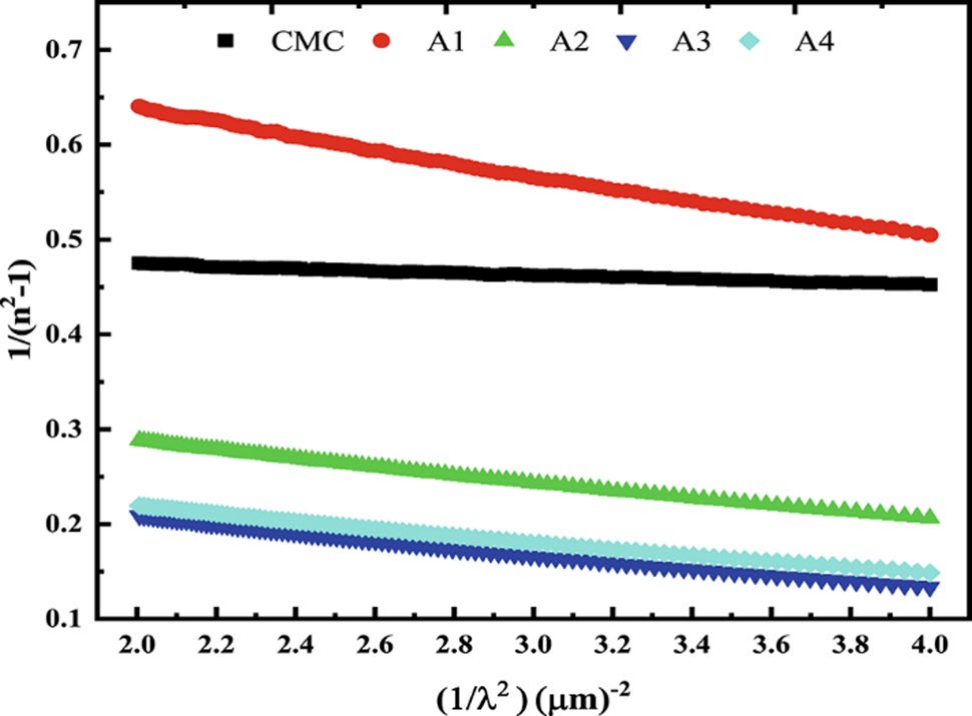
Plot of 1/(n^2^−1) versus 1/λ^2^ of CMC and CMC doped with 2, 4, 6, and 8 wt% of ZnO-NPs. The linear fit was used to determine the high-frequency dielectric constant (ε_∞_) and to analyze the optical dispersion behavior of the films.

The values of E_o_ and E_d_ were determined from the slope and intercept of the linear plot of (n^2^−1)^−1^ versus (hν)^2^, as shown in Fig. 9. The high-frequency dielectric constant (ε_∞_) was obtained by extrapolating the linear portion of (n^2^−1)^−1^ versus 1/λ^2^ as presented in Fig. 10.

It was noted that as the concentration of ZnO-NPs increased, E_o_ decreased, indicating that the energy gap became more concentrated, allowing for low-energy electronic transitions to occur. On the other hand, a higher ZnO content is associated with a stronger E_d_, suggesting that electronic polarization effects are stronger. There was an observed rise in the high-frequency dielectric constant ε_∞_ as the concentration of ZnO increased^25,27,67^.

Additional characteristics including the free carrier concentration (N) and lattice dielectric constant (ε_l_) were taken into account in order to have a better understanding of the films’ physical properties. The relationship between the wavelength (λ) and the real part of the dielectric constant is as follows:

14 $${\boldsymbol{n}}^{{\mathbf{2}}}={\boldsymbol{\varepsilon}}_{\boldsymbol{{\mathbf{l}}}}-\left(\frac{{\boldsymbol{e}}^{{\mathbf{2}}}\boldsymbol{N}}{4{\boldsymbol{\pi}}^{{\mathbf{2}}}{\boldsymbol{\varepsilon}}_{\boldsymbol{o}}{\boldsymbol{m}}^{\mathbf{*}}{\boldsymbol{c}}^{{\mathbf{2}}}}\right){\boldsymbol{\lambda}}^{{\mathbf{2}}}$$

where ε_l_, denotes the lattice dielectric constant, e denotes the elementary charge, ε_o_ denotes the vacuum permittivity, and N/m* denotes the ratio of free carrier concentration to the effective mass of carriers. This relation allows the determination of carrier-related optical parameters from the refractive index dispersion.

**Fig. 11:**
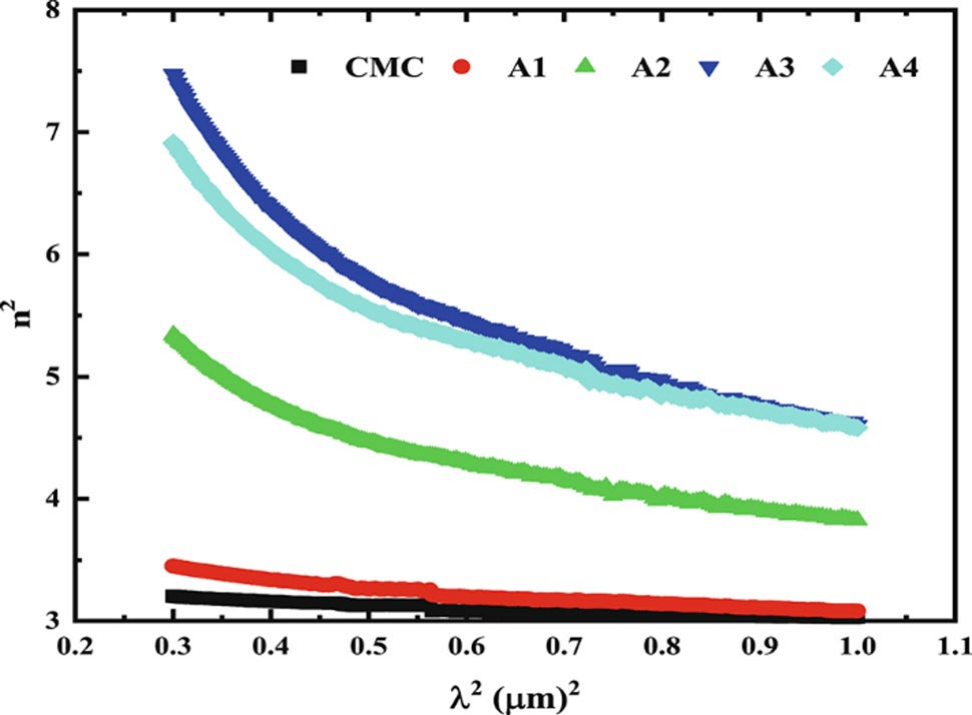
Linear relationship between n^2^ and λ^2^ of CMC and the CMC doped with different concentrations of ZnO. The slope and intercept of the linear fit were used to calculate the lattice dielectric constant (ε_l_) and the ratio of free carrier concentration to effective mass (N/m*).

Figure 11 illustrates the linear correlation between n² and λ². The slope and intercept of the fitted linear regression were employed to determine the lattice dielectric constant (ε_l_) and the ratio of free carrier concentration to effective mass (N/m*). The computed values are presented in Table 5. It was determined that ε_l_ exceeds the high-frequency dielectric constant (ε_∞_), signifying that free charge carriers influence the dielectric response at infinite frequency. The lattice dielectric constant rises with the concentration of ZnO-NPs up to 6 wt%. Table 5 indicates that the N/m* ratio is comparatively high owing to the minimal effective mass of electrons. Moreover, N/m* escalates with the augmentation of ZnO content, indicating that the films become increasingly richer in crystalline constituents as the concentration of ZnO-NPs grows.

**Table 5:** Optical dispersion parameters of pure CMC and CMC/ZnO nanocomposite films with varying ZnO-NPs concentrations.

ZnO Wt.%	Absorption parameter		Dispersion parameter						
	Onset gap (eV)	HOMO-LUMO gap (eV)	*n*	E_O_ (eV)	E_d_ (eV)	$\varepsilon_{\infty}$	$\varepsilon_{l}$	*N*/M* (g^−1^cm^− 3^)	*N* (g^−1^cm^3^)
0	–	5.21	1.74	5.00	9.32	3.01	3.16	4.41 × 10^55^	4.02 × 10^28^
1	–	5.10	1.63	4.24	8.11	2.27	3.39	9.93 × 10^55^	9.05 × 10^28^
2	2.61	4.67	1.84	3.58	9.46	3.66	4.88	3.39 × 10^56^	3.09 × 10^29^
3	2.58	4.43	1.94	3.23	10.99	4.45	6.49	6.03 × 10^56^	5.50 × 10^29^
4	2.39	3.98	2.30	3.39	11.38	4.40	6.12	4.88 × 10^56^	4.45 × 10^29^

### UV-blocking capacity

Three bands of the sun’s ultraviolet (UV) spectrum are known as UVC, UVB, and UVA, ranging 200–280 nm, 280–320 nm, and 320–400 nm, respectively. Protecting ourselves from the UV radiation is crucial since it can cause photo-oxidation, photo-carcinogenesis, and photo-aging. Utilizing a UV-Vis spectrophotometer operating within the 200–1000 nm wavelength range, the transmittance of films composed of CMC and ZnO-NPs was measured.

**Fig. 12:**
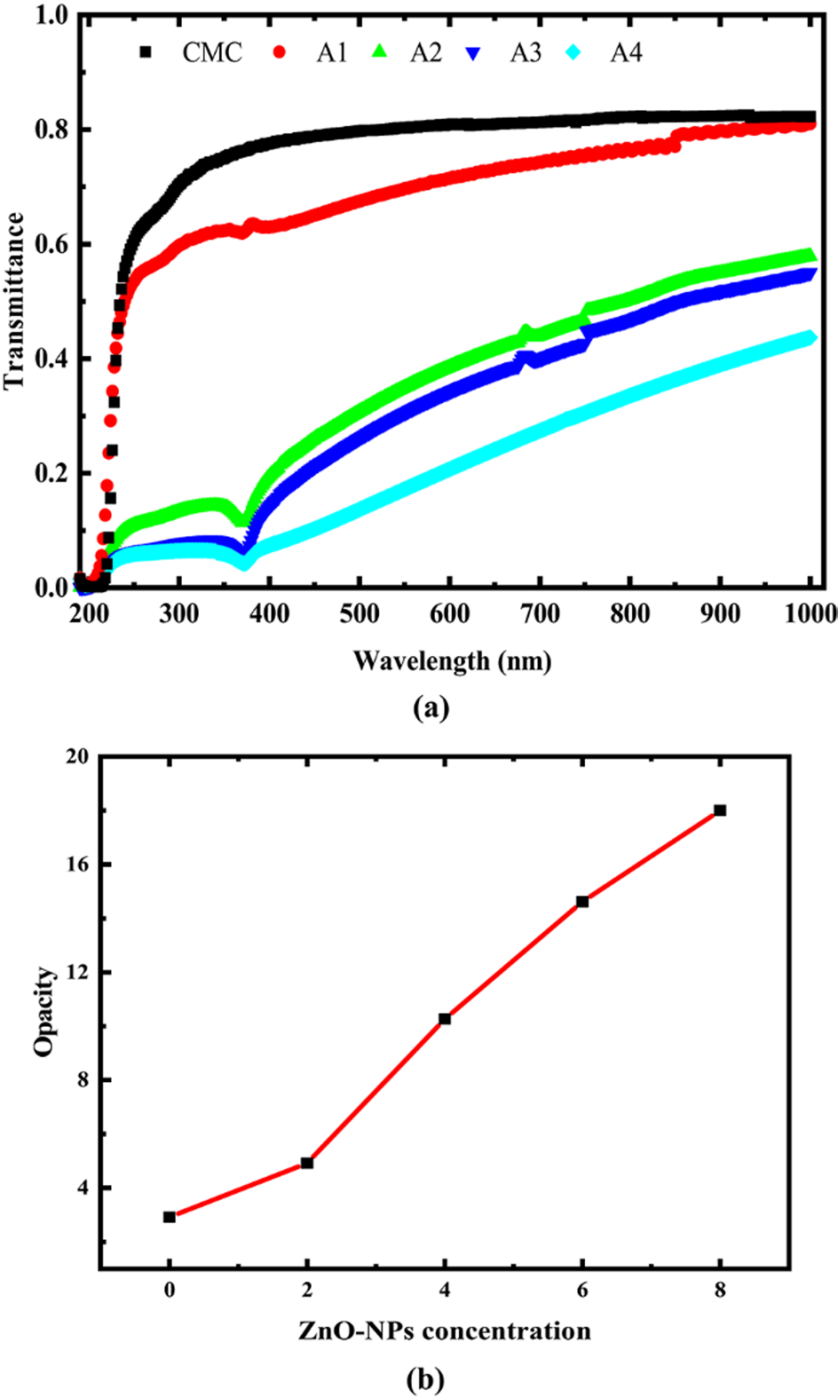
(**a**) UV–Vis transmittance spectra of CMC and the CMC doped with different concentrations of ZnO; (**b**) Variation of film opacity at 600 nm with ZnO concentration.

Figure 12a displays the transmittance spectrum of CMC and ZnO based nanocomposite films. A high UV transmittance of 0.17–66% in UVC, 66–73% in UVB, and 73–77% in UVA is exhibited by pure CMC. The UV transmittance is decreased upon incorporation of ZnO-NPs. For instance, a negligible decrease in UV transmission is noted with 2 wt% ZnO. Reduced transmittance of 0.21–12% for UVC, 12–14% for UVB, and 14–19% for UVA is achieved at 4 wt% ZnO. With a 6 wt% increase in ZnO content, the transmission of UVC drops to 0.2–7%, UVB to 7–8%, and UVA to 8–14%. Further reductions to 0.5–6% in UVC, 6–6.5% in UVB, and 6.5–7% in UVA transmittance are observed at 8 wt% ZnO, respectively. UVA radiation is subdivided into UVA2 (320–340 nm) and UVA1 (340–400 nm). Achieving complete blockage in the UVA1 spectrum poses difficulties for sunscreen applications. CMC/ZnO films provide optimal UVA1 protection at 8 wt% ZnO, exhibiting transmittance of roughly 6% at 340 nm and 7% at 400 nm, in contrast to pure CMC transmittance values of 74% and 77% at the corresponding wavelengths. It is observed that the spectral edge of CMC’s transmittance curve continuously shifts towards longer wavelengths as ZnO content increases, signifying a decrease in the optical bandgap. Factors influencing composite transmittance encompass particle size, refractive index, film thickness, filler content, surface roughness, and particle dispersion. Particles larger than the visible wavelength scatter light, diminishing transparency, but well-dispersed nanoparticles effectively absorb UV light. Ultraviolet blocking transpires through both absorption and scattering, as elucidated by Rayleigh theory^66^.

The significant drop in optical transmittance of the CMC/ZnO nanocomposite films cannot be attributable to absorption alone. Although ZnO is well known for its capacity to absorb UV radiation, the presence of nanoparticles inside the polymer matrix may add to light loss via scattering mechanisms. Although scattering was not directly assessed in this investigation, UV-blocking efficacy is likely due to a mix of absorption and scattering. This clarification was included to improve the accuracy of evaluating the samples’ optical behaviour.

Table 6 lists the UV-blocking percentages for the UVC, UVB, and UVA spectra. The findings definitely demonstrate that UV-blocking efficacy increases with the concentration of ZnO-NPs, with the 8 wt% ZnO film offering nearly total UV protection. Figure 12b illustrates the correlation between opacity and ZnO content at 600 nm. Opacity was determined utilizing:

15 $$\boldsymbol{O}\boldsymbol{p}\boldsymbol{a}\boldsymbol{c}\boldsymbol{i}\boldsymbol{t}\boldsymbol{y}=\frac{{\boldsymbol{A}}_{{\mathbf{600}}}}{\boldsymbol{t}}$$

A_600_ denotes the film absorbance at 600 nm, whereas t represents the film thickness in millimeters. The findings demonstrate that the sample with 8 wt% of ZnO displays the greatest opacity. Films with elevated ZnO content exhibit enhanced opacity, signifying diminished transparency.

**Table 6:** UV-blocking percentages in the UVC, UVB, and UVA regions for pure CMC film and CMC/ZnO nanocomposite films with different ZnO concentrations.

CMC loaded with ZnO-NPs (wt%)	UV—blocking %		
	UVC	UVB	UVA
0	34	27–34	23–27
2	43	39–43	37–39
4	88	86–88	81–88
6	93	92–93	86–92
8	94	93.5–94	93–93.5

The data presented in Table 7 clearly demonstrate the interdependence between the optical band gap energy and the UV-blocking efficiency of the CMC-based nanocomposite films. A general trend can be observed where films with a lower band gap exhibit superior UV-shielding performance. This relationship can be attributed to the enhanced light absorption capability in the UV region, as materials with narrower band gaps can absorb photons of lower energy, thereby preventing their transmission through the film.

Among the studied systems, the CMC/CuO@ZnO hybrid nanocomposite displayed the smallest band gap value of 0.83 eV and nearly completes blocking of UVC, UVB, and UVA radiation^26^. This exceptional behavior arises from the synergistic interaction between CuO and ZnO in the core/shell configuration, which facilitates interfacial charge transfer, reduces electron–hole recombination, and broadens the optical absorption range. Similarly, the CMC/CuO nanocomposite exhibited a low band gap (1.13 eV) and excellent UV protection, confirming the strong absorption ability of transition metal oxides with narrow electronic band structures^25^.

Conversely, graphene oxide (GO)-based nanocomposites, such as CMC/CuO/GO and CMC/ZnO/GO, exhibited higher band gap energies and comparatively weaker UV-blocking efficiencies as reported by Badry, R^28^. This can be explained by the partial transparency of GO sheets, which enhance film uniformity and electrical conductivity but reduce the density of localized electronic states responsible for strong UV absorption.

The CMC/ZnO nanocomposites developed in the present study shows an intermediate performance, combining a moderate band gap value of 2.39 eV with high UV-blocking capability of > 93%. This indicates that ZnO-NPs, though possessing a wider intrinsic band gap (~ 3.3 eV), can effectively interact with the CMC matrix through surface states and defect levels, extending the absorption toward the UV region.

Overall, the comparison highlights that the optical and UV-shielding properties of CMC-based films are strongly influenced by the type, morphology, and electronic structure of the embedded nano-fillers. The UV-blocking performance differences observed in Table 7 arise from variations in nanofiller dispersion, polymer–filler interfacial interactions, and microstructural evolution within CMC-based films. Additionally, nano-fillers are known to alter polymer chain packing, free volume, and crystallinity parameters that strongly influence optical absorption and band-gap behavior. Systems with fillers that disrupt chain ordering or increase defect density typically exhibit improved UV attenuation because defects and localized states enhance photon absorption pathways. Accordingly, the synergistic design of hybrid nanostructures, such as CuO@ZnO, appears to be the most effective strategy for achieving superior UV protection and tailored optoelectronic performance.

**Table 7:** Comparison of UV-blocking ability of different CMC-based nanocomposite films.

Film Type	Concentration (wt%)	$E_{g}^{in}$ (eV)	UV-Blocking %			Reference
			UVC (%)	UVB (%)	UVA (%)	
CMC/CuO	8	1.13	100.0	99.0	99.0	^21^
CMC/CuO@ZnO	4	0.83	99.9	99.9	99.9	^22^
CMC/CuO/GO	4	3.43	100.0	89.0	80.0	^23^
CMC/ZnO/GO	4	4.94	80.0	74.0	66.0	^23^
CMC/ZnO	8	2.39	94.0	93.5	93.0	Present study

### Evaluation of optical property stability over time

The absorbance and transmittance values of the pure CMC and CMC doped with 8wt.% of ZnO-NPs were re-measured after more than 6 months of storage under ambient conditions. The thickness of the new segments of the pure CMC and A4 are 0.034 and 0.02 mm, respectively. The results showed that the values remained closely consistent, indicating excellent long-term stability of the optical properties. This supports the reliability of the samples for practical applications.

**Fig. 13:**
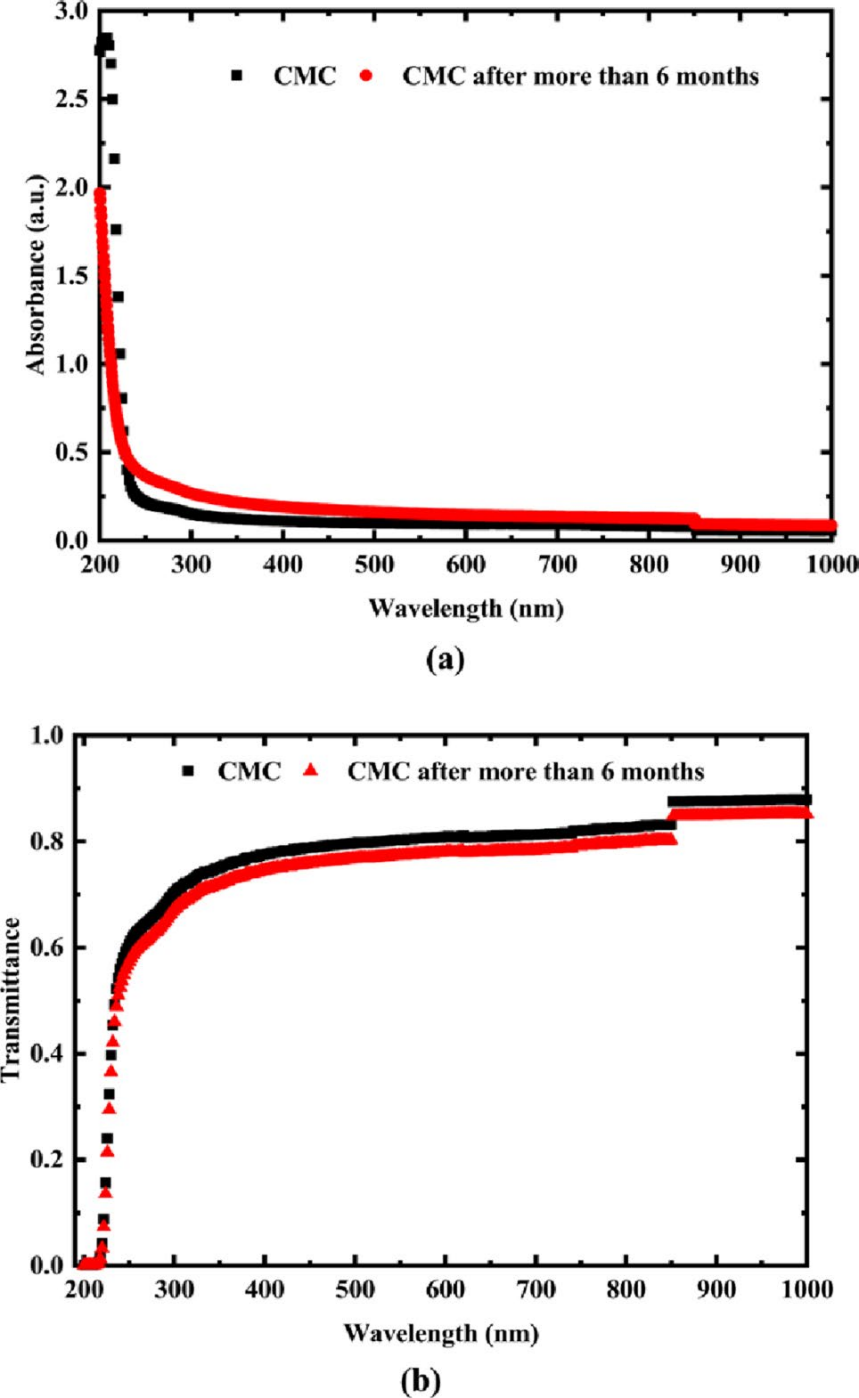

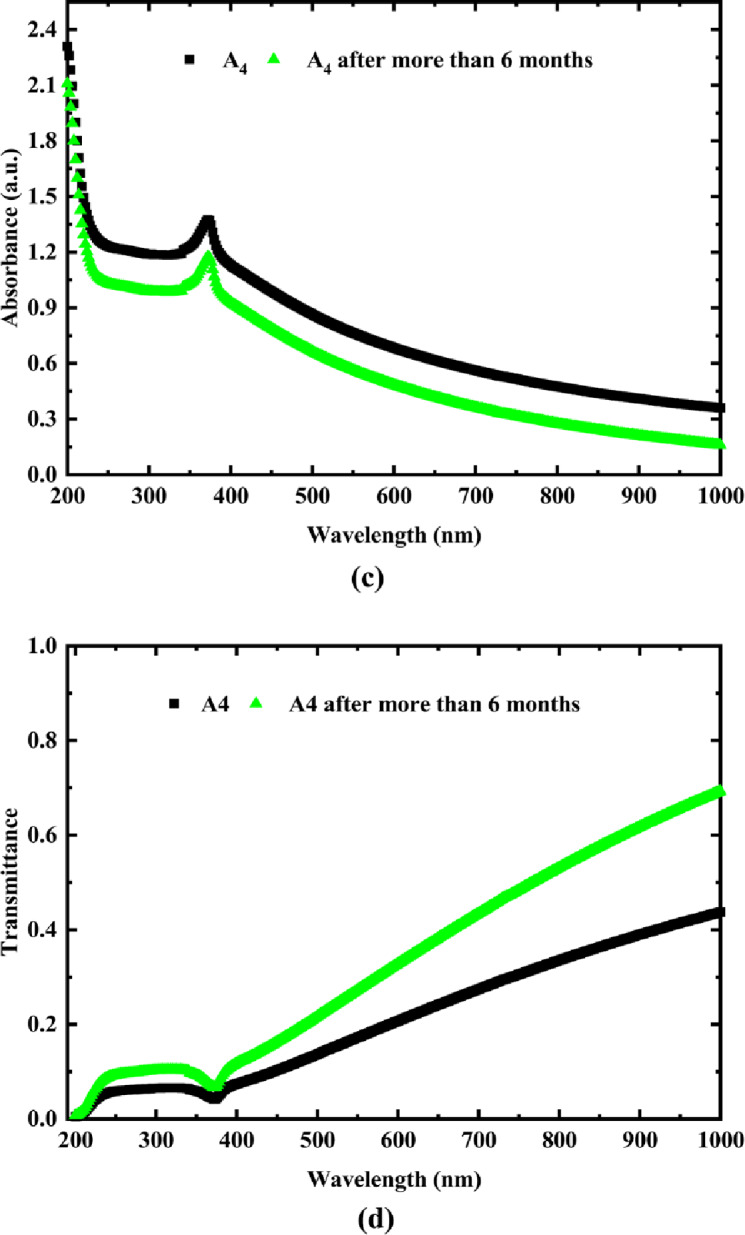
UV-Vis absorbance and transmittance spectra of pure CMC and CMC filled with 8 wt% of ZnO-NPs at the first time of measurement and after more than 6 months.

Figure 13 shows the absorbance and transmittance spectra of pure CMC and CMC doped with 8wt.% of ZnO-NPs. The overall absorption pattern of pure CMC and sample A4 remained consistent upon re-measurement, with only minor differences observed in absorbance intensity. Other studies report that polymeric systems can produce almost identical spectra with only slight intensity changes while remaining structurally stable^68^.

In our case, the observed difference in intensity can be attributed to an increase in the thickness of the sample fragment used for measurement. A thicker optical path naturally leads to higher absorbance, which aligns with the expected relationship between path length and measured intensity. Therefore, the consistency in spectral shape, along with a small change in intensity, can be explained by the difference in sample thickness rather than any alteration in the optical properties or chemical stability of the CMC.

It is important to note that CMC is a highly hydrophilic polymer, and its tendency to absorb moisture may influence both the optical response and structural stability of the CMC/ZnO films. Although the present study did not experimentally evaluate the effect of environmental humidity or moisture uptake on the optical performance, this factor represents a relevant limitation and should be considered in future investigations, particularly for practical applications where the films may be exposed to variable humidity conditions.

### Packaging test with green chillies

Although the synthesized films exhibited improved physical characteristics, a preliminary evaluation using green chillies as a model food was carried out to examine their moisture-retention performance under UVA light. In green chillies, weight loss mainly results from water evaporation and nutrient degradation. Water loss leads to surface wrinkling and shrinkage in the chillies, which diminishes their natural gloss and firmness. As moisture continues to decrease, the pulp becomes harder and less flexible, resulting in a decline in freshness and visual quality and ultimately reducing the chillies’ commercial value.

**Fig. 14:**
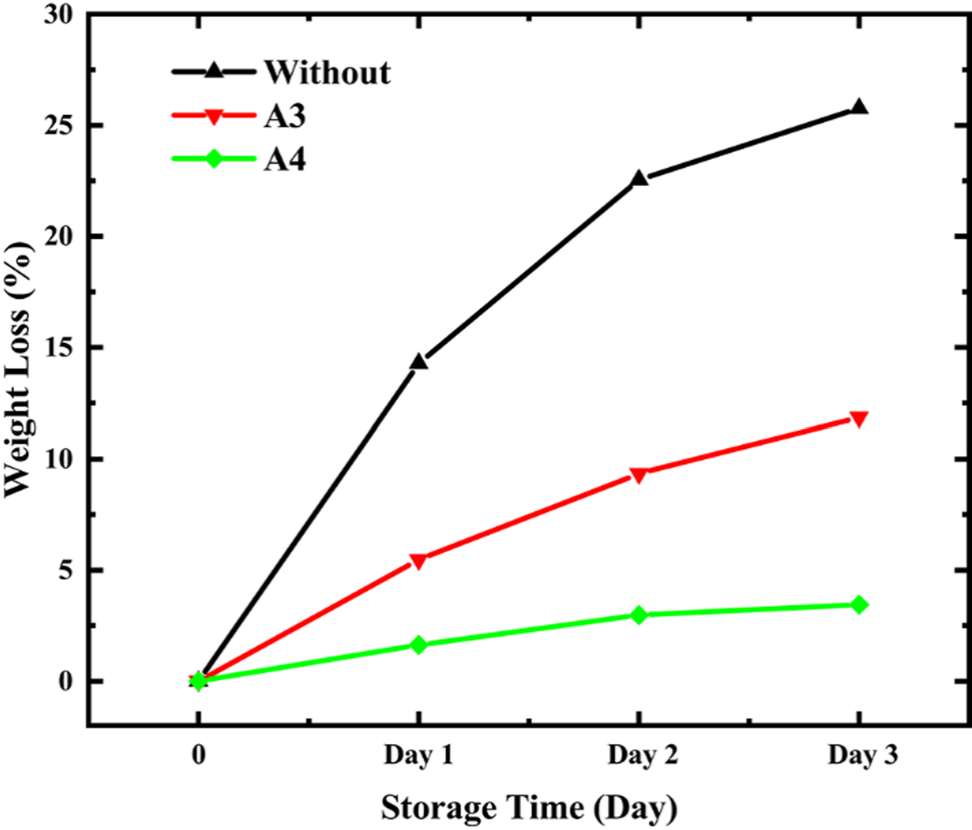
Weight loss rate of green chilies packaged with CMC/ZnO nanocomposite films containing 6 and 8 wt.% of ZnO-NPs for 3 days.

Evaluating the weight loss of green chilies in relation to the packaging material is essential, as it plays a key role in maintaining their freshness. To investigate this effect, the weight changes of chilies stored without any film and those packaged with the CMC/ZnO nanocomposite films were recorded over a 3 day period under exposure to UVA light, as presented in Fig. 14.

The weight reduction of green chillies was assessed after one to 3 days of storage. The weight loss of green chillies was calculated using Eq. 16

16 $$\bf Weight Loss\%= \Big[\Big(\frac{m_{i} - m_{f}}{m_{i}}\Big)\Big]\times 100$$

In this expression, m_i_ (g) denotes the initial weight of the fresh green chillies, whereas m_f_ (g) represents the weight of the chillies after the specified storage period. As illustrated in Fig. 14, the unpackaged sample exhibited a weight loss of 25.78% after 3 days of storage. In comparison, the sample packaged with the A3 film showed a lower weight loss of 11.87%. However, incorporating 8wt.% ZnO into the CMC matrix further decreased moisture loss, with the A4 sample achieving the lowest weight loss at 3.44%.

**Fig. 15:**
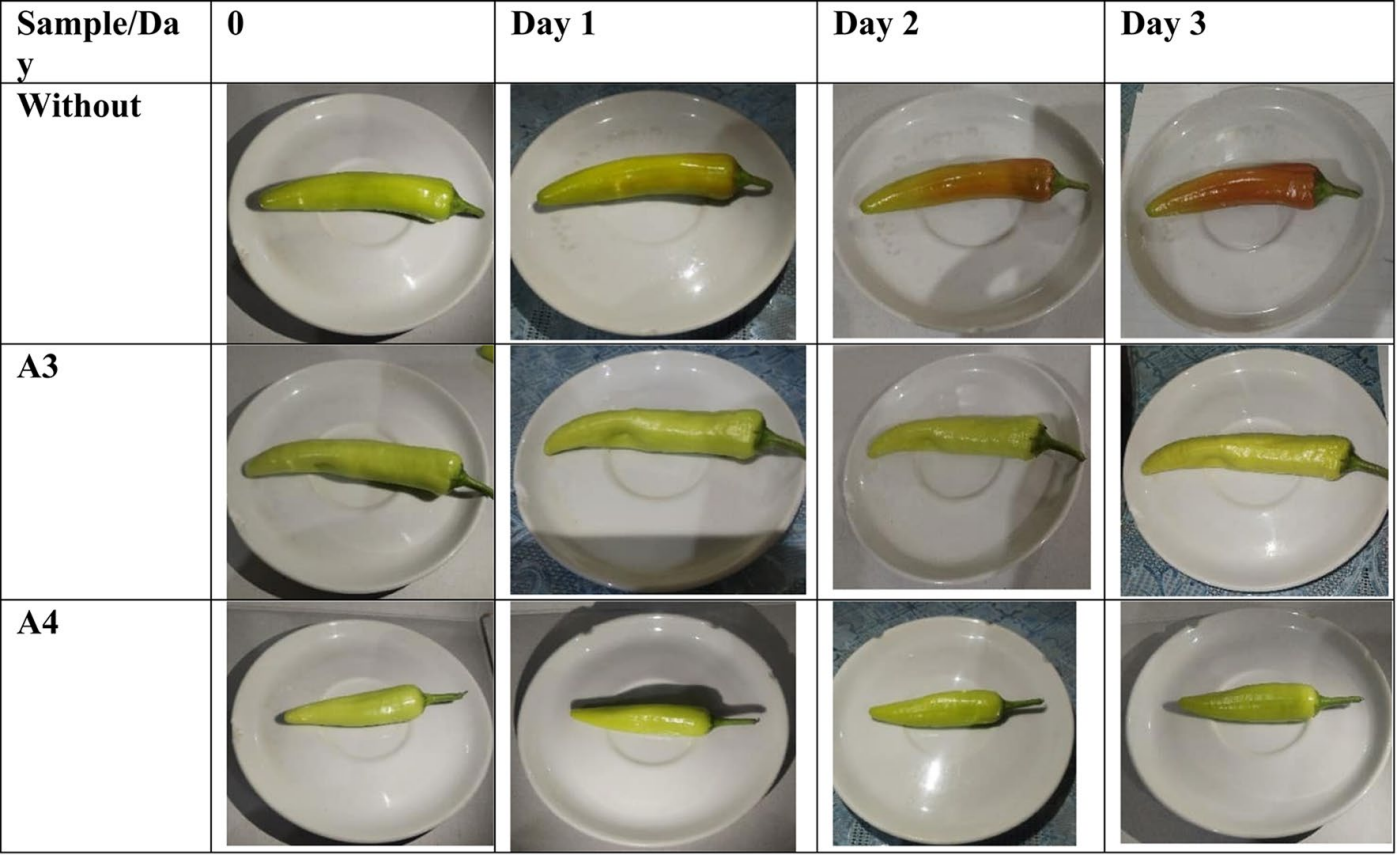
Food preservation test of green chillies (**a**) without packaging and packaging in (**b**) A3 and (**c**) A4 nanocomposite bags.

The images of the green chilli samples at both the start and the end of the experiment illustrate noticeable physical changes. In the control group without packaging (Fig. 15), pronounced wrinkling and surface shrinkage were observed. Additionally, the color of the chilli changed from green to orange and then to red with increased exposure to UV light from one to 3 days. These changes resulted from substantial weight loss combined with exposure to external environmental factors such as moisture, light, and temperature.

The samples packaged with A3 film exhibited noticeably fewer surface wrinkles compared with the previously described case. Notably, the sample wrapped with the A4 film retained nearly its original appearance after 3 days, showing no evident spoilage or substantial wrinkling. These observations align with the results obtained from the weight-loss analysis.

The green chilli test gives an initial visual indication of the moisture-retaining ability of the film. While the observed weight loss reduction is encouraging, this result should be considered preliminary. More extensive analyses are needed to establish the film suitability for commercial food contact use. These include microbial resistance testing, oxygen permeability and food contact migration limits to ensure full compliance with safety and performance requirements.

## Conclusion

Using a solution casting-technique aided by ultrasonication, CMC/ZnO nanocomposite films with varying ZnO weight fractions were produced. The films’ structural and optical properties were investigated using Fourier transform infrared (FTIR) and X- ray diffraction (XRD) analysis. Analysis of the UV-Vis optical data revealed several optical properties for both pure CMC and CMC/ZnO nanocomposites. These included refractive index, absorption coefficients, extinction coefficients, dispersion energy parameters, and optical dielectric constants. Tauc and ASF models were used to evaluate the optical band gap energy of the nanocomposites, and the Urbach energies were also calculated. Results showed that compared to pure CMC, the band gap energies of the nanocomposites decreased as the proportion of ZnO-NPs in the composite structure increased. These results indicate that incorporating ZnO-NPs into the CMC matrix influences its optical properties, suggesting enhanced interaction between the polymer chains and ZnO-NPs. The optical band gap energies were lowered because the nanocomposites showed an increase in free carriers and interband localized energy states. In addition, higher refractive indices, lower single-oscillator energies, and larger dispersion energies were all outcomes of the increase in the ZnO concentration. Free charge carriers were responsible for the consistently greater lattice dielectric constant compared to the high-frequency dielectric constant. An increase in the Urbach energy was observed as the ZnO content rose, suggesting that the nanocomposite exhibited enhanced localized states. Additionally, the optical transmittance in the UVC, UVB, and UVA areas decreased dramatically as the ZnO content increased, indicating high UV-blocking ability of the films. The UV-blocking efficiency of the 8 wt% ZnO film was 94% in the UVC range, 93.5% in the UVB range, and 93% in the UVA range. These results confirm that the CMC/ZnO nanocomposites offer tunable optical properties, making them promising candidates for various optoelectronic applications. Additionally, the optical results of the CMC/CuO, CMC/CuO@ZnO, CMC/CuO/GO, and CMC/ZnO/GO nanocomposites indicates that the minimum bandgap values and the maximum UV-shielding capacity arise when CMC is combined with an individual metal oxide or core/shell structure of two metal oxides, which yields lower gaps than those obtained by introducing graphene oxide with the metal oxide. The results confirm that pure CMC and ZnO-NP-doped CMC films exhibit excellent long-term stability, with negligible changes in absorbance and transmittance after storage for a long time. This stability indicates that the prepared films are suitable for applications requiring durable and reliable optical behavior over time. The packaging trial with green chillies demonstrated the films’ potential to reduce moisture loss, addressing the common deterioration signs of wrinkling and reduced gloss caused by water evaporation. These findings highlight the films’ applicability in food packaging systems where maintaining freshness and physical integrity is essential under UVA exposure.

## Data Availability

The datasets used and/or analyzed during the current study are available from the corresponding author upon reasonable request.
